# Bioactive Diketopiperazines and Nucleoside Derivatives from a Sponge-Derived *Streptomyces* Species

**DOI:** 10.3390/md17100584

**Published:** 2019-10-16

**Authors:** Lamiaa A. Shaala, Diaa T. A. Youssef, Jihan M. Badr, Steve M. Harakeh, Grégory Genta-Jouve

**Affiliations:** 1 Natural Products Unit, King Fahd Medical Research Center, King Abdulaziz University, Jeddah 21589, Saudi Arabia; 2 Department of Medical Laboratory Technology, Faculty of Applied Medical Sciences, King Abdulaziz University, Jeddah 21589, Saudi Arabia; 3 Suez Canal University Hospital, Suez Canal University, Ismailia 41522, Egypt; 4 Department of Natural Products, Faculty of Pharmacy, King Abdulaziz University, Jeddah 21589, Saudi Arabia; jihanbadr2010@hotmail.com; 5 Department of Pharmacognosy, Faculty of Pharmacy, Suez Canal University, Ismailia 41522, Egypt; 6 Special Infectious Agents Unit, King Fahd Medical Research Center, King Abdulaziz University, Jeddah 21589, Saudi Arabia; sharakeh@gmail.com; 7 UMR 8038 CiTCoM, Faculté de Pharmacie de Paris, Université Paris Descartes, Avenue de l’observatoire, 75006 Paris, France; gregory.genta-jouve@parisdescartes.fr; 8 Molecules of Communication and Adaptation of Microorganisms (UMR 7245), National Museum of Natural History, CNRS, 75231 Paris, France

**Keywords:** sponge-derived *Streptomyces* sp. Call-36, diketopiperazine alkaloids, nucleoside derivatives, NMR and ECD calculations, cytotoxic and antimicrobial activities

## Abstract

Fractionation and purification of the ethyl acetate extract of the culture of a sponge-derived actinomycete, *Streptomyces* species Call-36, resulted in the isolation and identification of a new diketopiperazine, actinozine A (**1**), cyclo(2-OH-d-Pro-l-Leu) (**2**), two new nucleosides, thymidine-3-mercaptocarbamic acid (**3**) and thymidine-3-thioamine (**4**), together with cyclo(d-Pro-l-Phe) (**5**) and cyclo(l-Pro-l-Phe) (**6**). The structure assignments of the compounds were carried out by interpretation of 1D and 2D NMR data and mass spectral determinations. The absolute configurations of **1** and **2** were determined by Marfey’s method and by comparison of the experimental and TDDFT-calculated ECD spectra. Actinozine A possesses an unprecedented hydroperoxy moiety at C-2 of the proline moiety, while **3** and **4** possess unusual mercaptocarbamic acid and thiohydroxylamine functionalities at *N*-3 of the thymine moiety. The isolated compounds displayed variable cytotoxic and antimicrobial activities.

## 1. Introduction

Secondary metabolites played a vital role in discovery and development of drugs and saved the lives of millions of people over the past decades [1,2]. Microbial secondary metabolites contributed significantly to drug discovery and pharmaceutical industry. There is a continuous need for novel chemical entities with new mechanisms of action to overcome drug resistance and to fulfill the medical needs for new drugs [3]. This need forced the chemists to search for understudied microbial resources that inhabit challenging environments [4]. The marine environment, with its great biological diversity, has been considered as under-investigated source of microbes with unlimited biosynthetic capabilities for bioactive compounds [4]. Marine actinobateria, including actinomycetes, have proven to be producers of novel chemical entities with diverse bioactivities such as antitumor, antimicrobial, anti-inflammatory, antiparasitic, antimalarial, antiviral, antioxidant, anti-angiogenesis and many others [5,6]. The 2,5-diketopiperazines (DKPs) belong to the smallest cyclic dipeptides that consist of a six-membered ring containing two amide linkages where the two nitrogen atoms and the two carbonyls are at opposite positions. DKPs are represented in structurally diverse groups of secondary metabolites, with different examples showing significant bioactivities including antimicrobial, antitumor, analgesic, and many other biomedical indications [7]. Prominent anticancer DKPs include the anti-microtubule phenylahistins [8], the cell cycle inhibitors tryprostatins [9], the chaetocins, which inhibit the lysine-specific histone methyltransferase [10] and the ardeemins with their reversal effects on multiple drug resistant (MDR) phenotype [11].

**Figure 1:**
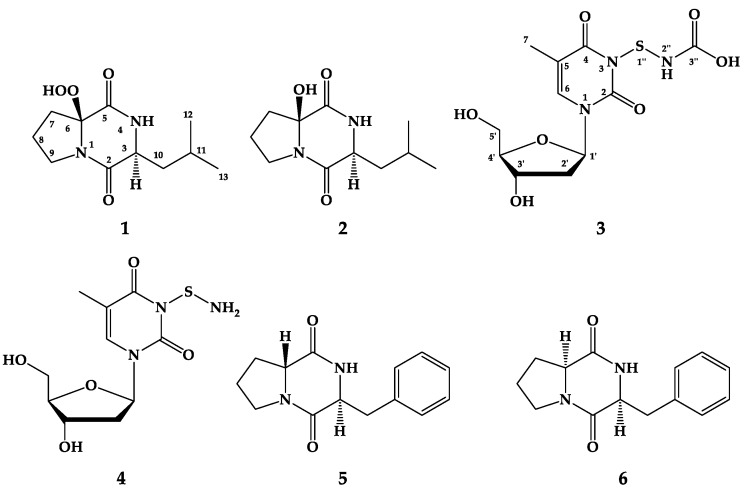
Structures of compounds **1**–**6**.

As a part of ongoing search on marine microbial secondary metabolites [12,13,14,15,16,17], the ethyl acetate extract of the sponge-derived *Streptomyces* species Call-36 was studied. A new diketopiperazine alkaloid with a hydroperoxy group at C-2 of the proline moiety, actinozine A (**1**), cyclo(2-OH-Pro-l-Leu) (**2**) [18], two new nucleoside derivatives, thymidine-3-mercaptocarbamic acid (**3**) and thymidine-3-thioamine (**4**), in addition to the previously reported compounds cyclo(d-Pro-l-Phe) (**5**) [19] and cyclo(l-Pro-l-Phe) (**6**) [20] (Figure 1) were isolated. The structure assignments of the compounds were supported by comprehensive investigations of the NMR and MS data. The relative configuration of **1** and **2** was determined using the DP4 probability approach. The absolute configuration of the amino acid moieties in **1** and **2** were established by Marfey’s method and by comparison of both experimental and predicted ECD spectra. In this paper, the structure determinations as well as the cytotoxic and antimicrobial activities of the compounds are discussed.

## 2. Results and Discussion

### 2.1. Purification of the Compounds 

The ethyl acetate extract of the fermentation broth of the actinomycete, *Streptomyces* sp. Call-36, was subjected to extensive chromatographic separations over silica gel, Sephadex LH-20 and final HPLC purification to yield compounds **1**–**6**.

### 2.2. Structure Elucidation of the Compounds 

**Figure 2:**
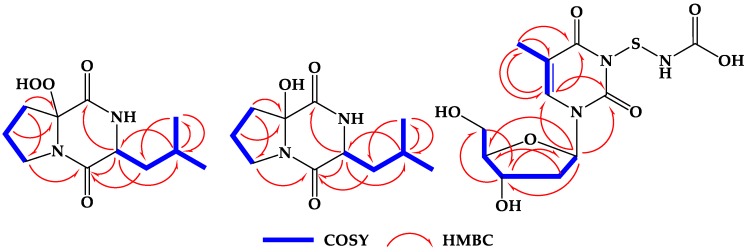
Key COSY and HMBC correlations of **1**, **2**, and **3**.

Compound **1** (Figure 1) was isolated as a colorless solid with molecular formula C_11_H_18_N_2_O_4_. The NMR spectra of **1** (Figures S1–S6 and Table 1) displayed 11 signals including two methyls, four methylenes, two methines, and three quaternary carbons (two carbonyls and one oxygenated carbon) as supported by the ^13^C NMR and HSQC experiments (Table 1). The ^1^H and ^13^C NMR spectra of **1** (Figures S1 and S2 and Table 1) displayed characteristic signals for a diketopiperazine ring, which included two amidic carbonyls at δ_C_ 168.0 (qC, C-2) and 167.2 (qC, C-5), a methine signal at δ_H_/δ_C_ 4.04 (dt, 7.2, 3.0 Hz, H-3)/56.0 (CH, C-3) and an amidic singlet at δ_H_ 6.25 (1H, brs, NH). In addition, the remaining ^1^H and ^13^C signals of **1** supported the presence of leucine and 2-substituted proline units. The presence of ^1^H-^1^H spin-spin coupling system in the COSY experiment of **1** (Figure S4) from H_2_-7 to H_2_-9 supported this assignment (Figure 2). The interruption of the coupling system at C-6 suggested its quaternary nature. The NMR signals at δ_H_/δ_C_ 167.2 (qC, C-5), 95.5 (qC, C-6), 2.44, 2.15 (H_2_-7)/33.4 (CH_2_, C-7), 2.16, 2.08 (H_2_-8)/19.2 (CH_2_, C-8), and 3.90, 3.62 (H_2_-9)/45.3 (CH_2_, C-9) are characteristic for a 2-substituted proline residue. In addition, the presence of a leucine unit was supported from the NMR signals at δ_H_/δ_C_ 168.0 (qC, C-2), 4.04 (H-3)/56.0 (CH, C-3), 6.25 (4-NH), 1.96, 1.84 (H_2_-10)/44.1 (CH_2_, C-10), 1.80 (H-11)/24.5 (C-11), 0.99 (H_3_-12)/23.1 (C-12) and 0.95 (H_3_-13)/21.1 (C-13). Again, the presence of a second ^1^H-^1^H spin-spin coupling system in the COSY experiment between H-3 and the isobutyl moiety at C-3 (H-10 → H_3_-13) supported the presence leucine unit (Figure 2). Despite the similarity between the NMR data of **1** with those of **2** (Table 2), there is a significant downfield shift of C-6 in **1** by 8.8 ppm (resonating at δ_C_ 95.5 instead of δ_C_ 86.7 in **2**) (Table 2), suggesting the presence of a hydroperoxy (OOH) (δ_H_ 9.27, brs) group at C-6 in **1** instead of OH in **2** (Table 2). Finally, all protonated carbons were assigned from HSQC (Figure S6) and COSY experiments (Figure 2 and Figure S4), while the placement of the OOH moiety at C-6 was supported by HMBC correlations of H_2_-7/C-6 and H_2_-8/C-6 (Table 1, Figure 2 and Figure S6). Similarly, the assignment of the quaternary amidic carbons (C-2 and C-5) was secured from HMBC correlations (Table 1, Figure 2 and Figure S6). The relative configuration at C-6 was determined by comparison between experimental and theoretical NMR chemical shifts as proposed by Smith and Goodman [21] as several recent applications of this approach demonstrated to be efficient [22,23]. 

**Figure 3:**
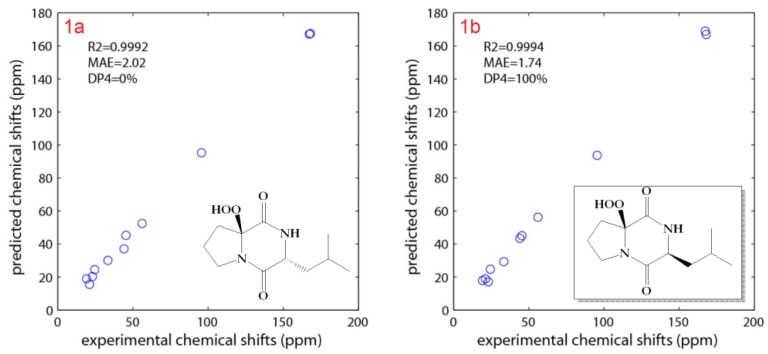
Calculated and experimental NMR chemical shifts of **1**.

**Figure 4:**
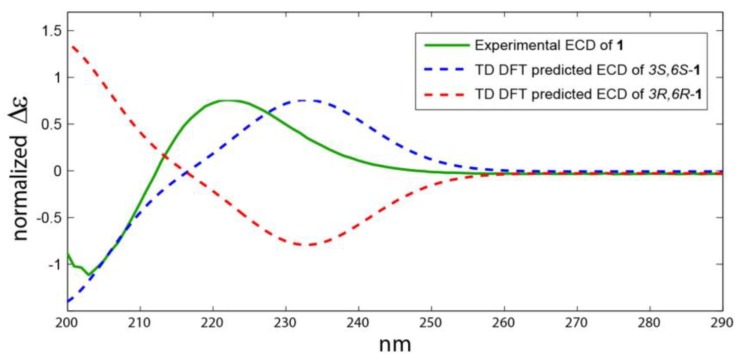
Experimental and predicted ECD spectra of **1**.

As shown on Figure 3, the 3S*,6S* relative configuration for **1b** was supported by all the metrics used. The correlation between experimental and theoretical chemical shifts was higher for **1b** (R^2^ = 0.9994) and the mean average error (MAE) value was lower for **1b** and finally a 100% DP4 score for **1b** (Figure 3). All calculations for both isomers (3S,6S and 3R,6R) of **1** can be found at (https://figshare.com/s/d626b72364548b03e11b). The absolute configuration of the leucine residue was determined by Marfey’s method [24]. HPLC analysis of the derivative of 1-fluoro-2,4-dinitrophenyl-5-l-alanine amide (FDAA, Marfey’s reagent) with the hydrolytic products of **1** gave the same retention time as the derivative prepared from an authentic l-leucine, and therefore the l-configuration was assigned to the leucine residue. Due to the hydrolysis of **1** under acidic condition, the absolute configuration at C-6 was not determined by Marfey’s method. The absolute configuration at C-3 and C-6 asymmetric centers was determined from analysis of the ECD spectrum of **1**. Comparison between experimental and theoretical ECD spectra gave a very good agreement as displayed on Figure 4. Two Cotton effects with alternative signs were observed due to the n→π* transition (Figure 4). Thus, **1** was assigned as (3S,8aS)-8a-hydroperoxy-3-isobutylhexahydropyrrolo[1,2-a]pyrazine-1,4-dione {cyclo(2-OOH-d-Pro-l-Leu } and is reported here as a new natural product and named actinozine A.

**Figure 5:**
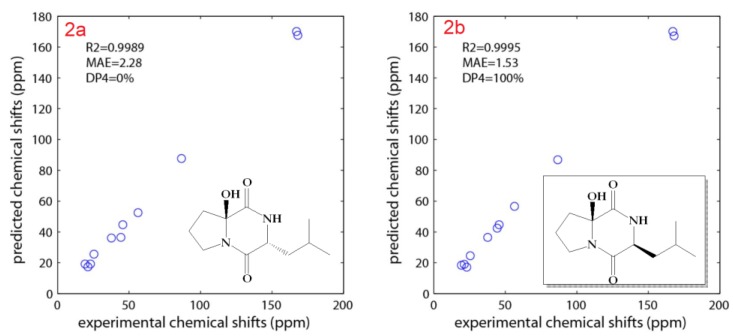
Calculated and experimental NMR chemical shifts of **2**.

**Figure 6:**
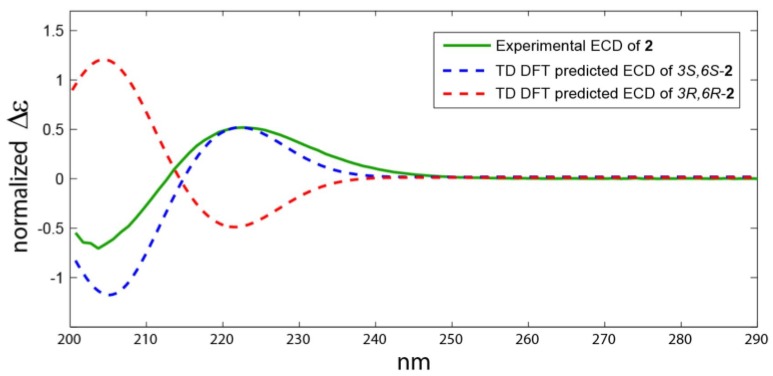
Experimental and predicted ECD spectra of **2**.

Compound **2** (Figure 1) was purified as colorless oil with molecular formula of C_11_H_18_N_2_O_3_. The combined one- and two-dimensional NMR data of **2** (Table 2, Figure 2 and Figures S8–S13) are in good agreement with those reported for cyclo(2-OH-Pro-l-Leu) [18]. However, the absolute configuration at C-6 was missing. For the determination of the relative configuration, we followed the same approach as described above. As expected, the same 3S* and 6S* was obtained with a high probability (R^2^ = 0.9995) (Figure 5). All calculations for both isomers (3S,6S and 3R,6R) of **2** can be found at (https://figshare.com/s/d626b72364548b03e11b). Again, the absolute configuration of the leucine residue was determined by Marfey’s method [24] as discussed above and was found to be l-Leucine. Attempts to determine the stereochemistry at C-6 by Marfey’s method was not successful because of the decomposition of **2** under acidic conditions. Therefore, a CD spectrum for compound **2** (Figure 6) was recorded in order to determine the absolute configuration of the hydroxylated C-6. The absolute configuration at C-3 and C-6 in **2** was determined to be 3S and 6S by comparison of the experimental and predicted ECD spectra (Figure 6). Thus, **2** was assigned as cyclo(2-OH-d-Pro-l-Leu) {(3S,8Sa)-8a-hydroxy-3-isobutylhexahydropyrrolo[1,2-a]pyrazine-1,4-dione}. 

**Figure 7:**
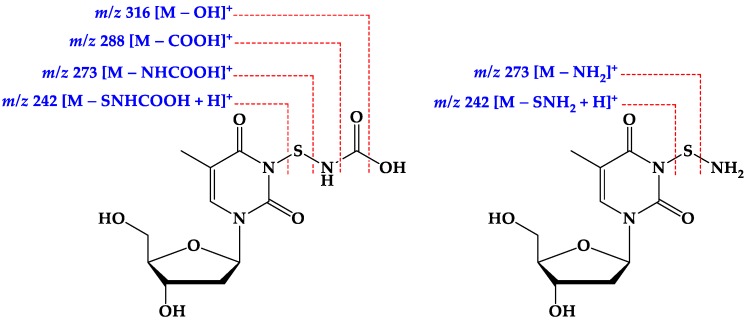
Key MS/MS fragmentation ion peaks of **3** and **4**.

**Figure 8:**
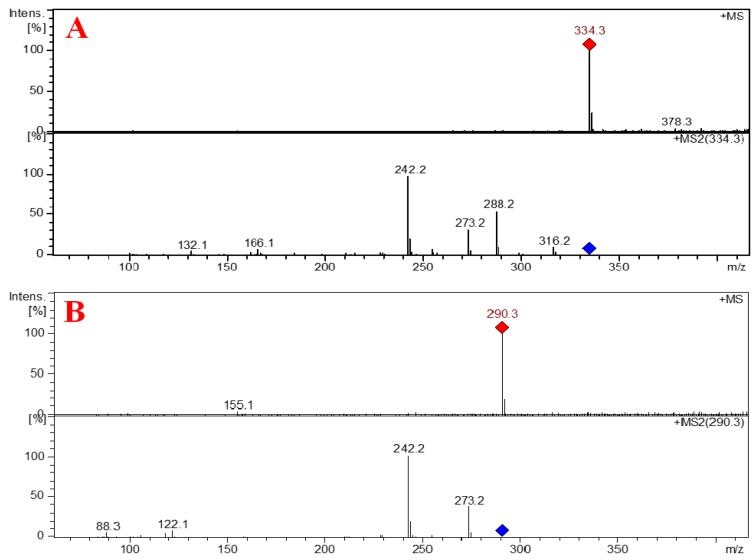
MS/MS fragmentation spectra of **3** (**A**) and **4** (**B**).

**Figure 9:**
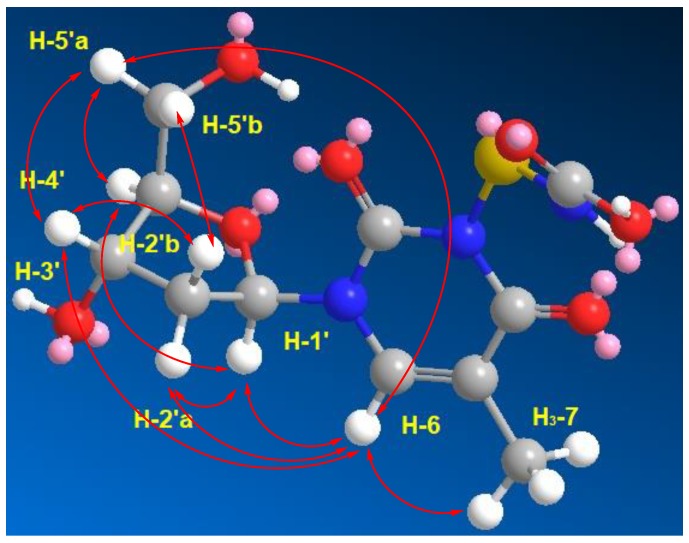
Observed ^1^H-^1^H ROESY correlations for **3**.

Compound **3** (Figure 1) was isolated as a white solid with molecular formula of C_11_H_15_N_3_O_7_S as established from the HRESIMS pseudomolecular ion peak at *m/z* 334.0708 [M + H]^+^, suggesting six degrees of unsaturation. The ^1^H and ^13^C NMR spectrum of **3** (Table 3 and Figures S15 and S16) showed signals for 2′-deoxyribose unit at δ_H_/δ_C_ 6.15 (1H, t, *J* = 7.0 Hz, H-1′)/83.7 (CH, C-1′), 2.07 (1H, m, H-2′a) and 2.02 (1H, m, 2′b)/39.5 (CH_2_, C-2′), 4.22 (1H, quin, *J* = 3.0 Hz, H-3′)/70.3 (CH, C-3′), 5.24 (1H, brs, O*H*-3′), 3.74 (1H, q, *J* = 3.5 Hz, H-4′)/87.2 (CH, C-4′) and 3.57 (1H, dd, *J* = 12.0, 3.5 Hz, H-5′a), 3.53 (1H, dd, *J* = 12.0, 4.0 Hz, H-5′b)/61.3 (CH_2_, C-5′) and 5.05 (1H, brs, O*H*-5′). This was supported by the presence of a continuous ^1^H-^1^H spin-spin coupling system in the COSY experiment (Figure S17) from H-2′ to H_2_-5′ (Figure 2), ^1^*J*_CH_ correlations in the HSQC experiment (Figure S18) and HMBC correlations (Table 3, Figure 2 and Figure S19). The NMR data of the 2′-deoxyribose moiety (Table 3) are in good agreement with the literature [25,26,27]. In addition, the signals at δ_H/C_ 150.4 (qC, C-2), 163.7 (qC, C-4), 109.3 (qC, C-5), 7.68 (1H, s, H-6)/136.1 (CH, C-6), 1.75 (3H, s, H_3_-7)/12.2 (CH_3_, C-7) supported the presence of a substituted thymine moiety in **3** [25,26]. Again, the COSY and HMBC correlations in **3** (Table 3 and Figure 2) supported the presence of a thymine moiety. The remaining portion of **3** includes the elemental composition of CH_2_NO_2_S (S-NH-COOH), which corresponds to a mercaptocarbamic acid (Figure 1). The existence of the mercaptocarbamic acid moiety was supported by the presence of NMR signals at δ_H/C_ 7.26 (1H, brs, N*H*) and 10.90 (1H, hump, H-3′′)/158.1 (qC, C-3′′) (Table 3) and by MS/MS fragmentation ion peaks at *m*/*z* 316 [M − OH]^+^, 288 [M − COOH]^+^, 273 [M − NHCOOH]^+^ and 242 [M − SNHCOOH + H]^+^ (Figure 7 and Figure 8), completing the degrees of unsaturation and the molecular formula of **3**. The ^13^C chemical shift of the carboxylic acid moiety of the carbamic acid is in good agreement with the literature [28]. The placement of the 2′-desoxyribose moiety at *N*-1 was supported by HMBC long-range correlations from H-1′ to C-2 and C-6 (Table 3 and Figure 2). Since *N*-3 is the only available and free place in **3**, thus, the mercaptocarbamic acid moiety was linked to *N*-3 to complete the degrees of unsaturation and the planner structure of **3**. The strong ROESY correlation (Figure S20) displayed between H-1′ and H-4′ protons and the absence of any ROESY correlations between H-3′ and H-1′ as well as H-3′ and H-4′ strongly supported the *β* configuration of the nucleoside (Table 3 and Figure 9). Detailed additional and expected ROESY correlations in **3** are presented in Table 3 and Figure 9. The similarity of the value and sign of the optical rotation of **3** (+11°) with those of d-thymidine (+18.5°) [29] supported the d-sugar moiety in **3**. Accordingly, **3** was assigned as thymidine-3-mercaptocarbamic acid and is reported here as a new natural product. 

Compound **4** (Figure 1) was purified as a white powder with molecular formula of C_10_H_15_N_3_O_5_S as established by the HRESIMS pseudomolecular ion peak at *m/z* 290.0812 [M + H]^+^, being 44 mass unit less than **3**. Comparison of the ^1^H and ^13^C NMR data of **4** with those of **3** (Table 4 and Figures S23 and S24) displayed identical signals, suggesting similar structures of both compounds. The protonated and quaternary carbons were assigned from 2D NMR experiments including COSY, HSQC and HMBC experiments (Figures S25–S27). The loss of 44 mass unit in **4** suggested the absence of the COOH moiety (Table 4). The presence of a thiohydroxylamine moiety in **4** was also supported by MS/MS fragmentation ion peaks at *m/z* 273 [M − NH_2_]^+^ and 242 [M − SNH_2_ + H]^+^ (Figure 7 and Figure 8), completing the planner structure of **4**. The configuration of the sugar moiety of **4** was established by comparison of the NMR data of **3** and **4** (Table 3 and Table 4). In addition, both compounds displayed positive optical rotation values (+11° and +13°, respectively), supported the same *β* configuration of the sugar part and the d-nucleoside in both compounds. Therefore, **4** was assigned as thymidine-3-thioamine and is reported here as a new natural product. It is worth to mention that, the occurrence of the mercaptocarbamic acid and thiohydroxylamine moieties in **3** and **4** are presented here for the first time from a natural source.

Compounds **5** and **6** were identified by interpretation of their NMR (Figures S29–S38) and MS data as well as their optical rotations and by comparison with available data in the literature. The absolute configuration of the amino acid moieties in **5** and **6** was determined by HPLC analysis of the derivatives of 1-fluoro-2,4-dinitrophenyl-5-l-alanine amide (FDAA, Marfey’s reagent) with the hydrolytic products of **5–7** as previously reported [24]. Accordingly, the compounds were assigned as cyclo(d-Pro-l-Phe) (**5**) [19] and cyclo(l-Pro-l-Phe) (**6**) [20]. 

The compounds were evaluated for their cytotoxic activities in the sulforhodamine B (SRB) assay [30] against HCT-116 (colorectal carcinoma, ATCC CCL-247) and MCF-7 (breast cancer, ATCC HTB-22). Compound **5** showed moderate activity with IC_50_ of 32.7 µM against HCT-116, while other compounds were weakly active against this cell line. On the other hand, all compounds showed weak activities against breast cancer cell line (Table 5). 

Furthermore, the antimicrobial activities of the compounds were evaluated against *S. aureus* and *C. albicans* using disc diffusion assay [31] at 100 μg/disc. Compounds **1** and **2** were potently active against *S. aureus* with inhibition zones of 23 and 20 mm, respectively, while these compounds displayed moderate activity against *C. albicans* with inhibition zones of 19 and 16 mm, respectively (Table 5). On the other hand, compounds **5** and **6** displayed moderate activity against *S. aureus* and *C. albicans* with inhibition zones of 9.0–14.0 mm (Table 5). 

As a result, the moderate cytotoxicity of **5** and the weak cytotoxicity of other diketopiperazines (**1**, **2**, and **6**) may suggest the need to screen these compounds against a variety of other cell lines or completely different biological target. Moreover, the strong antimicrobial activities of **1** and **2** support the use of such compounds as a template or scaffold to develop structurally related antimicrobial drugs.

## 3. Materials and Methods

### 3.1. General Experimental Procedures 

Optical rotations were measured on a JASCO DIP-370 digital polarimeter at 25 °C at the sodium D line (589 nm). UV spectra were recorded on a Hitachi 300 spectrometer (Hitachi High-Technologies Corporation, Kyoto, Japan). The ECD spectra were obtained on a JASCO J-810 spectropolarimeter with a 0.5 cm cell in MeOH. IR spectra were measured on a Shimadzu Infrared-400 spectrophotometer (Shimadzu, Kyoto, Japan). 1D and 2D NMR spectra (chemical shifts in ppm, coupling constants in Hz) were recorded on Bruker Avance DRX 600 MHz (600 MHz for ^1^H and 150 MHz for ^13^C NMR) (Bruker, Rheinstetten, Germany) and Bruker Ascend™ 850 MHz (850 MHz for ^1^H and 213 MHz for ^13^C NMR) (Bruker BioSpin, Billerica, MA, USA) spectrometers using CDCl_3_ or DMSO-*d*_6_ as solvent. NMR spectra were referenced to the residual protonated solvent signals (for CHCl_3_: 7.26 ppm for ^1^H and 77.0 ppm for ^13^C; for DMSO: 2.51 ppm for ^1^H and 39.6 ppm for ^13^C). Positive ion HRESIMS data were obtained with a Micromass Q-ToF equipped with leucine enkaphalin lockspray, using *m*/*z* 556.2771 [M + H]^+^ as a reference mass. For column chromatography, silica gel (Merck, 70-230 mesh ASTM) and Sephadex LH-20 (0.25–0.1 mm, Pharmacia) were used. Precoated silica gel 60 F-254 plates (Merck) were used for TLC. HPLC purifications were performed on a semi-preparative HPLC column (RP18, 5 μm, ARII Cosmosil, 250 × 10 mm, Waters). 

### 3.2. Biological Materials 

The actinomycete *Streptomyces* species Call-36 was isolated from the Red Sea sponge *Callyspongia* species. To purify the actinomycete strain from the sponge materials, the previously reported protocol from our laboratory was used [16]. Identification of the actinomycete strain was based on 16S rRNA gene sequence analysis. Purification of the genomic DNA, the amplification of the 16S rRNA gene by PCR, and sequence alignment of the strain were performed as reported before [32]. The 16S rRNA gene sequence of the strain displayed 99% similarity with type strains of *Streptomyces albus* subsp. *albus* (AB184781) and *Streptmyces almquistii* (AB184258).

### 3.3. Fermentation and Extraction 

The spores of *Streptomyces* sp. Call-36 were cultured in Erlenmeyer flasks (2 L) containing 500 mL of ISP-2 fermentation media. The media contained 4.0 g of yeast extract, 10 g of malt extract and 4.0 g of dextrose and 3.3% sea salt in 1 L distilled water (pH 7.2). The cultures were incubated on a rotatory shaker at 180 rpm at 28 °C for 15 days. The combined fermentation broths (10 L) were partitioned against EtOAc and the resulting EtOAc solutions were concentrated under vacuum to give a brown residue (2.4 g).

### 3.4. Purification of the Compounds 

The EtOAc extract (2.4 g) was subjected to SiO_2_ VLC using n-hexane/CH_2_Cl_2_/MeOH gradients to give five fractions (A–E). Fraction C (370 mg) was purified by gel filtration over Sephadex LH-20 with MeOH to give four subfractions (C1-C4). Fraction C2 (120 mg) was purified on C18 HPLC column with 40% CH_3_CN to yield **1** (3.5 mg) and **2** (2.3 mg). Fraction C4 (60 mg) was purified on C18 HPLC column with 30% CH_3_CN to yield **3** (1.8 mg) and **4** (1.5 mg). Fraction D (410 mg) was purified by gel filtration over Sephadex LH-20 with MeOH to give five subfractions (D1-D5). Fraction D3 (110 mg) was further purified on C18 HPLC column with 40% CH_3_CN to yield **5** (2.5 mg) and **6** (3.6 mg). 

### 3.5. Spectral Data of the Compounds

Actinozine A (**1**): colorless solid; [α]_D_ −52° (*c* 0.1, MeOH); UV (MeOH) *λ*_max_ (log *ε*): 230 (4.15), 303 (4.09) nm; ECD (MeOH) [Δε]_203nm_ −15.40, [Δε]_224nm_ +13.27; IR (film) *ν*_max_ 3450, 1656, 1627 cm^−1^; NMR data: Table 1; HRESIMS *m*/*z* 243.1344 (calcd for C_11_H_19_N_2_O_4_, [M + H]^+^, 243.1345). 

Cyclo(2-OH-d-Pro-l-Leu) (**2**): colorless oil; [α]_D_ −50° (*c* 0.1, MeOH); UV (MeOH) *λ*_max_ (log *ε*): 231 (4.17), 305 (4.00) nm; ECD (MeOH) [Δε]_203nm_ −30.25, [Δε]_223nm_ +29.82; IR (film) *ν*_max_ 3445, 1659, 1628 cm^−1^; NMR data: Table 2; HRESIMS *m*/*z* 227.1395 (calcd for C_11_H_19_N_2_O_3_, [M + H]^+^, 227.1396). 

Thymidine-3-mercaptocarbamic acid (**3**): white solid; [α]_D_ +11° (*c* 0.1, MeOH); UV (MeOH) *λ*_max_ (log *ε*): 265 (3.45), 255 (3.50) nm; NMR data: Table 3; HRESIMS *m*/*z* 334.0708 (calcd for C_11_H_16_N_3_O_7_S, [M + H]^+^, 334.0709). 

Thymidine-3-thioamine (**4**): white solid; [α]_D_ +13° (*c* 0.1, MeOH); UV (MeOH *λ*_max_ (log *ε*): 266 (3.50), 256 (3.55) nm; NMR data: Table 3; HRESIMS *m*/*z* 290.0812 (calcd for C_10_H_16_N_3_O_5_S, [M + H]^+^, 290.0811). 

### 3.6. Determination of the Configuration of the Leucine Moiety in **1** and **2**

Compounds **1** and **2** (each about 0.5 mg) were heated separately in 1 mL of 6 N HCl at 100 °C for 16 h, followed by removal of the excess HCl under vacuum. To each dry hydrolysate, 200 µL of 1% solution of 1-fluoro-2,4-dinitrophenyl-5-l-alanine amide (FDAA, Marfey’s reagent) [24] in acetone and 40 µL of 1.0 M NaHCO_3_ were added. The reaction mixture was heated at 45 °C for 1.5 h, cooled, and acidified with 20 µL of 2.0 M HCl. Similarly, standard amino acids (d and l) of phenylalanine and leucine were derivatized separately. The derivatized standard amino acids and hydrolysates of **1** and **2** were subjected to HPLC on Nova-Pak C18 reverse-phase column (150 × 3.9 mm i.d., 4 mm particle size; Waters, Milford, MA, USA) using the following gradient program. Solvent A was a 50 mM triethylamine–phosphate buffer (pH 3.5) containing 25% (v/v) MeOH, and solvent B was the same buffer containing 70% MeOH. The mobile phase was a linear gradient from 0 to 100% B (100 to 0% A) in 40 min, at a flow rate of 0.65 mL/min at 25 °C. The eluted peaks were monitored at 340 nm. The retention times for FDAA derivatives of standards and compounds **1** and **2** were as follows: (l)-leucine (*t*_R_ 27.2 min), (d)-leucine (*t*_R_ 36.0 min), compound **1** (*t*_R_ 27.2 min) and compound **2** (*t*_R_ 27.2 min) (Figure S39).

### 3.7. Computational Details

All DFT calculations have been performed using Gaussian 16 [33]. A conformation analysis was conducted using the GMMX plugin followed by a geometry optimization at the B3LYP/6-31g(d) level. A frequency check was performed at the same level of theory. GIAO NMR properties were calculated at the mpw1pw91/6-311+g(2d,p) level. DP4 probabilities were calculated using our own implementation of the algorithm published by Smith and Goodman [21]. Rotational strengths were calculated on 20 excited states using the b3lyp/6-31g(d) level of theory. The ECD spectra were plotted using Gaussview 6. All calculations for both isomers of **1** and **2** (3S,6S and 3R,6R) can be found at (https://figshare.com/s/d626b72364548b03e11b).

### 3.8. Cytotoxicity Evaluation 

The cytotoxicity of the compounds was evaluated against three tumorous human cell lines including colorectal carcinoma (HCT-116, ATCC CCL-247) and breast cancer (MCF-7, ATCC HTB-22) (Figure S40). The evaluation of the cytotoxicity of the compounds was carried out by sulforhodamine B (SRB) assay as reported before [30]. 

### 3.9. Antimicrobial Evaluation 

A disc diffusion assay was used to determine the antimicrobial activity of the compounds [31] with replication (*n* = 3). *Staphylococcus aureus* and *Candida albicans* served as target models for bacteria and fungi. A total of 100 µg of each compound was loaded onto 6-mm sterile circular filter-paper discs. The paper discs were left to air-dry. The dried paper discs were placed onto nutrient agar plates that had already been inoculated with a lawn of target microorganisms. After 24 h of incubation, the antimicrobial activity of the compounds was calculated.

## 4. Conclusions 

In conclusion, investigation of a sponge-derived actinomycete, *Streptomyces* sp. Call-36, yielded a new diketopiperazine alkaloid, actinozine A (**1**), with a hydroperoxy functionality at C-2 of the proline moiety, two new thymidine derivatives, thymidine-3-mercaptocarbamic acid (**3**) and thymidine-3-thioamine (**4**), with unprecedented functionalities at *N*-3 of the thymine moiety. Additionally, the previously reported compounds cyclo(2-OH-d-Pro-l-Leu) (**2**), cyclo(d-Pro-l-Phe) (**5**) and cyclo(l-Pro-l-Phe) (**6**) were isolated. The one- and two-dimensional NMR data and MS spectral determinations supported the assignment of the compounds. The absolute stereochemistry of **1** and **2** were established by Marfey’s method and by comparison of the experimental and TDDFT-calculated ECD spectra with the experimental ones. Compounds **1** and **2** displayed potent activity against *S. aureus* and were moderately active against *C. albicans*. Finally, compound **5** displayed moderate and selective activity against HCT-116 with an IC_50_ of 32.7 μM.

## Figures and Tables

**Table 1:** NMR data of compound **1** (600 and 150 MHz, CDCl_3_).

No.	δ_C_ (mult.)	δ_H_ [mult., *J* (Hz)]	HMBC
2	168.0, qC		
3	56.0, CH	4.04 (dt, 7.2, 3.0)	C-2, C-5, C-10, C-11
4 (N*H*)	-	6.25 (brs)	
5	167.2, qC		
6	95.5, qC		
7	33.4, CH_2_	2.44 (ddd, 13.2, 7.2, 3.0) 2.15 (m)	C-6, C-8, C-9
8	19.2, CH_2_	2.16 (m), 2.08 (m)	C-6, C-7, C-9
9	45.3, CH_2_	3.90 (ddd, 12.0, 8.6, 7.8) 3.62 (ddd, 12.0, 9.6, 4.2)	C-2, C-7, C-8
10	44.1, CH_2_	1.96 (m), 1.84 (t, 7.2)	C-2, C-3, C-12, C-13
11	24.5, CH	1.80 (nonet, 6.6)	C-3, C-12, C-13
12	23.1, CH_3_	0.99 (d, 6.6)	C-10, C-11
13	21.1, CH_3_	0.95 (d, 6.6)	C-10, C-11
OO*H*		9.27 (brs)	

**Table 2:** NMR data of compound **2** (600 and 150 MHz, CDCl_3_).

No.	δ_C_ (mult.)	δ_H_ [mult., *J* (Hz)]	HMBC
2	168.0, qC		
3	56.4, CH	3.97 (td, 10.4, 4.2)	C-2, C-5, C-10, C-11
4 (N*H*)	-	6.02 (brs)	
5	167.2, qC		
6	86.7, qC		
7	37.6, CH_2_	2.30 (m), 2.15 (m)	C-6, C-8, C-9
8	19.2, CH_2_	2.18 (m), 2.08 (m)	C-6, C-7, C-9
9	45.6, CH_2_	3.73 (m), 3.66 (m)	C-2, C-7, C-8
10	44.4, CH_2_	1.88 (dd, 10.2, 7.2) 1.80 (m)	C-2, C-3, C-12, C-13
11	24.5, CH	1.80 (m)	C-3, C-12, C-13
12	23.0, CH_3_	1.00 (d, 6.0)	C-10, C-11
13	21.2, CH_3_	0.94 (d, 6.0)	C-10, C-11
O*H*		2.97 (brs)	

**Table 3:** NMR data of compound **3** (600 and 150 MHz, DMSO-*d*_6_).

No.	δ_C_ (mult.)	δ_H_ [m., *J* (Hz)]	HMBC	ROESY
2	150.4, qC	-		
4	163.7, qC	-		
5	109.3, qC	-		
6	136.1, CH	7.68 (s)	C-2, C-4, C-7	H_3_-7, H-1′, H-2′a, H-4′, H-5′a
7	12.2, CH_3_	1.75 (s)	C-4, C-5, C-6	H-6
1′	83.7, CH	6.15 (t, 7.0)	C-2, C-6, C-2′, C-3′	H-2′a, H-4′, H-6
2′a 2′b	39.5, CH_2_	2.07 (m) 2.02 (m)	C-4′	H-1′, H-6 H-3′, H-4′, H-5′b
3′	70.3, CH	4.22 (quin, 3.0)	C-1′, C-5′	H-2′b
4′	87.2, CH	3.74 (q, 3.5)		H-2′b, H-5′a, H-6
5′a 5′b	61.3, CH_2_	3.57 (dd, 12.0, 3.5) 3.53 (dd, 12.0,4.0)	C-3′	H-4′, H-6 H-2′b
O*H*-3′		5.24 (brs)		
O*H*-5′		5.05 (brs)		
N*H*		7.26 (brs)		
3′′ (*C*OO*H*)	158.1, qC	10.90 (hump)		

**Table 4:** NMR data of compound **4** (850 and 213 MHz, DMSO-*d*_6_).

No.	δ_C_ (mult.)	δ_H_ [m., *J* (Hz)]	HMBC
2	150.4, qC	-	
4	163.7, qC	-	
5	109.3, qC	-	
6	136.1, CH	7.70 (s)	C-2, C-4, C-7
7	12.2, CH_3_	1.77 (s)	C-4, C-5, C-6
1′	83.7, CH	6.16 (t, 6.8)	C-2, C-6, C-2′, C-3′
2′a 2′b	39.5, CH_2_	2.09 (ddd, 13.6, 7.7, 6.0) 2.04 (ddd, 13.6, 6.0, 3.4)	C-4′
3′	70.4, CH	4.24 (m)	C-1′, C-5′
4′	87.2, CH	3.76 (q, 4.2)	
5′a 5′b	61.3, CH_2_	3.58 (brd, 12.0) 3.54 (brd, 12.0)	C-3′
O*H*-3′		5.26 (brs)	
O*H*-5′		5.06 (brs)	
N*H_2_*		^a^	

^a^ Not observed.

**Table 5:** Cytotoxic and antimicrobial activities of compounds **1**–**6**.

	IC_50_ (μM)		Inhibition Zones (mm) at 100 μg/disc	
Compound	HCT 116	MCF 7	*S. aureus*	*C. albicans*
1	146	88.8	23.0	19.0
2	150	117	20.0	16.0
3	≥50	90	NT	NT
4	≥50	112	NT	NT
5	32.7	81.9	14.0	11.0
6	131	123	10.0	9.0
Doxorubicin ^a^	1.62	0.77		
Ciprofloxacin ^b^			22.0	
Ketoconazole ^c^				30.0

NT = Not tested; ^a^ Positive cytotoxicity control; ^b^ Positive antibacterial control (5.0 µg/disc); ^c^ Positive antifungal control (50 µg/disc).

## Supplementary Materials

The Supplementary Materials are available online at https://www.mdpi.com/1660-3397/17/10/584/s1, Figure S1: ^1^H NMR spectrum of compound **1** (CDCl_3_), Figure S2: expansion of the ^1^H NMR spectrum of compound **1**, Figure S3: ^13^C NMR spectrum of compound **1** (CDCl_3_), Figure S4: ^1^H-^1^H COSY NMR spectrum of compound **1** (CDCl_3_), Figure S5: HSQC spectrum of compound **1** (CDCl_3_), Figure S6: HMBC spectrum of compound **1** (CDCl_3_), Figure S7: HRESIMS spectrum of compound **1**, Figure S8: ^1^H NMR spectrum of compound **2** (CDCl_3_), Figure S9: expansion of ^1^H NMR spectrum of compound **2** (CDCl_3_), Figure S10: ^13^C NMR spectrum of compound **2** (CDCl_3_)^,^ Figure S11: ^1^H-^1^H COSY NMR spectrum of compound **2** (CDCl_3_), Figure S12: HSQC spectrum of compound **2** (CDCl_3_), Figure S13: HMBC Spectrum of compound **2** (CDCl_3_), Figure S14: HRESIMS spectrum of compound **2**, Figure S15: ^1^H NMR spectrum and expansion of ^1^H NMR spectrum of compound **3** (DMSO-*d*_6_), Figure S16: ^13^C NMR spectrum of compound **3** (DMSO-*d*), Figure S17: ^1^H-^1^H COSY NMR Spectrum of compound **3** (DMSO-*d*), Figure S18: HSQC spectrum of compound **3** (DMSO-*d*_6_), Figure S19: HMBC spectrum of compound **3** (DMSO-*d*_6_), Figure S20: ROESY spectrum of compound **3** (DMSO-*d*_6_), Figure S21: HRESIMS spectrum of compound **3**, Figure S22: ^1^H NMR Spectrum of compound **4** (DMSO-*d*_6_), Figure S23: expansion of ^1^H NMR Spectrum of compound **4** (DMSO-*d*_6_), Figure S24: ^13^C NMR spectrum of compound **4** (DMSO-*d*_6_), Figure S25: ^1^H-^1^H COSY NMR Spectrum of compound **4** (DMSO-*d*_6_), Figure S26: HSQC Spectrum of compound **4** (DMSO-*d*_6_), Figure S27: HMBC spectrum of compound **4** (DMSO-*d*_6_), Figure S28: HRESIMS spectrum of compound **4**, Figure S29: ^1^H NMR spectrum of compound **5** (CDCl_3_), Figure S30: ^13^C NMR spectrum of compound **5** (CDCl_3_), Figure S31: ^1^H-^1^H COSY NMR spectrum of compound **5** (CDCl_3_), Figure S32: HSQC spectrum of compound **5** (CDCl_3_), Figure S33: HMBC spectrum of compound **6** (CDCl_3_), Figure S34: ^1^H NMR spectrum of compound **6** (CDCl_3_), Figure S35: ^13^C NMR Spectrum of compound **6** (CDCl_3_), Figure S36: ^1^H-^1^H COSY NMR spectrum of compound **6** (CDCl_3_), Figure S37: HSQC spectrum of compound **6** (CDCl_3_), Figure S38: HMBC spectrum of compound **6** (CDCl_3_), Figure S39: HPLC trace for Marfey’s-derivatized hydrolysates of compounds **1**,**2**,**5,6** and derivatized standard amino acids, Figure S40: Concentration-response profiles for compounds **1**–**6**.

## References

[1] Demain A.L., Zhang L., Zhang L., Demain A. (2005). Natural Products and Drug Discovery. Natural Products: Drug Discovery and Therapeutics Medicines.

[2] Zhang L., Zhang L., Demain A. (2005). Integrated Approaches for Discovering Novel Drugs from Microbial Natural Products. Natural Products: Drug Discovery and Therapeutics Medicines.

[3] Wright G.D., Sutherland A.D. (2007). New strategies for combating multidrug-resistant bacteria. Trends Mol. Med..

[4] Fenical W., Jensen P.R. (2006). Developing a new resource for drug discovery: Marine actinomycete bacteria. Nat. Chem. Biol..

[5] Manivasagana P., Venkatesana J., Sivakumar K., Kima S.K. (2014). Pharmaceutically active secondary metabolites of marine actinobacteria. Microbiol. Res..

[6] Blunt J.W., Carroll A.R., Copp B.R., Davis R.A., Keyzers R.A., Prinsep M.R. (2018). Marine natural products. Nat. Prod. Rep..

[7] Martins M., Carvalho I. (2007). Diketopiperazines: Biological activity and synthesis. Tetrahedron.

[8] Kanoh K., Kohno S., Katada J., Takahashi J., Uno I., Hayashi Y. (1999). Synthesis and biological activities of phenylahistin derivatives. Bioorg. Med. Chem..

[9] Cui C., Kakeya H., Okada G., Onose R., Osada H. (1996). Novel mammalian cell cycle inhibitors, tryprostatins A, B and other diketopiperazines produced by *Aspergillus fumigatus*. I. Taxonomy, fermentation, isolation and biological properties. J. Antibiot..

[10] Greiner D., Bonaldi T., Eskeland R., Roemer E., Imhof A. (2005). Identification of a specific inhibitor of the histone methyltransferase SU(VAR)3-9. Nat. Chem. Biol..

[11] Chou T., Depew K., Zheng Y., Safer M., Chan D., Helfrich B., Zatorska D., Zatorski A., Bornmann W., Denishefsky S. (1998). Reversal of anticancer multidrug resistance by the ardeemins. Proc. Natl. Acad. Sci. USA.

[12] Murshid S.S.A., Badr J.M., Youssef D.T.A. (2016). Penicillosides A and B: New cerebrosides from the marine-derived fungus *Penicillium* species. Rev. Bras. Farmacogn..

[13] Asiry I.A.M., Badr J.M., Youssef D.T.A. (2015). Penicillivinacine, antimigratory diketopiperazine alkaloid from the marine-derived fungus *Penicillium vinaceum*. Phytochem. Lett..

[14] Shaala L.A., Youssef D.T.A. (2015). Identification and bioactivity of compounds from the fungus *Penicillium* sp. CYE-87 isolated from a marine tunicate. Mar. Drugs.

[15] Mourshid S.A., Badr J.M., Risinger A.L., Mooberry S.L., Youssef D.T.A. (2016). Penicilloitins A and B, new antimicrobial fatty acid esters from a marine endophytic *Penicillium* species. Z. Naturforsch. C..

[16] Shaala L.A., Youssef D.T.A., Badr J.M., Harakeh S.M. (2016). Bioactive 2(1H)-pyrazinones and diketopiperazine alkaloids from a tunicate-derived actinomycete *Streptomyces* sp.. Molecules.

[17] Youssef D.T.A., Alahdal A.M. (2018). Cytotoxic and antimicrobial compounds from the marine-derived fungus, *Penicillium* species. Molecules.

[18] Nishanth S.K., Nambisan B., Dileep C. (2014). Three bioactive cyclic dipeptides from the *Bacillus* sp. N strain associated with entomopathogenic nematode. Peptides.

[19] Fdhila F., Vázquez V., Sánchez J.L., Riguera R. (2003). dd-Diketopiperazines: Antibiotics active against *Vibrio anguillarum* isolated from marine bacteria associated with cultures of *Pectenmaximus*. J. Nat. Prod..

[20] Munekata M., Tamura G. (1981). Selective inhibition of SV40-transformed cell growth by diketopiperazines. Agric. Biol. Chem..

[21] Smith S.G., Goodman J.M. (2010). Assigning stereochemistry to single diastereoisomers by GIAO NMR calculation: The DP4 Probability. J. Am. Chem. Soc..

[22] Roulland E., Solanki H., Calabro K., Zubia M., Genta-Jouve G., Thomas O.P. (2018). Stereochemical study of puna’auic acid, an allenic fatty acid from the Eastern Indo-Pacific cyanobacterium *Pseudanabaena* sp.. Org. Lett..

[23] Afoullouss S., Calabro K., Genta-Jouve G., Gegunde S., Alfonso A., Nesbitt R., Morrow C., Alonso E., Botana L.M.A., Louise Allcock A.L. (2019). Treasures from the deep: Characellides as anti-inflammatory lipoglycotripeptides from the sponge *Characella pachastrelloides*. Org. Lett..

[24] Marfey P. (1984). Determination of d-amino acids. II. Use of a bifunctional reagent, 1,5-difluoro-2,4-dinitrobenzene. Carlsberg Res. Commun..

[25] Hammoda H.M., Badr J.M., Youssef D.T.A. (2007). Three antioxidant compounds of the red alga *Liagora farinosa*. Nat. Prod. Sci..

[26] Abou-Hussein D.R., Badr J.M., Youssef D.T.A. (2007). Nucleoside constituents of the Egyptian tunicate *Eudistoma laysani*. Nat. Prod. Sci..

[27] Youssef D.T.A., Ibrahim S.R., Shaala L.A., Mohamed G.A., Banjar Z.M. (2016). New cerebroside and nucleoside derivatives from a Red Sea strain of the marine cyanobactrium *Moorea producens*. Molecules.

[28] Ethier A.L., Switzer J.R., Rumple A.C., Medina-Ramos W., Li Z., Fisk J., Holden B., Gelbaum L., Pollet P., Eckert C.A. (2015). The effects of solvent and added bases on the protection of benzylamines with carbon dioxide. Processes.

[29] https://www.sigmaaldrich.com/catalog/product/aldrich/855006?lang=en&region=SA.

[30] Vichai V., Kirtikara K. (2006). Sulforhodamine B colorimetric assay for cytotoxicity screening. Nat. Protoc..

[31] Kiehlbauch J.A., Hannett G.E., Salfinger M., Archinal W., Monserrat C., Carlyn C. (2000). Use of the National Committee for Clinical Laboratory Standards Guidelines for Disk Diffusion Susceptibility Testing in New York State Laboratories. J. Clin. Microbiol..

[32] Chun J., Goodfellow M. (1995). A phylogenetic analysis of the genus *Nocardia* with 16s rRNA gene sequences. Int. J. Syst. Bacteriol..

[33] Frisch M.J., Trucks G.W., Schlegel H.B., Scuseria G.E., Robb M.A., Cheeseman J.R., Scalmani G., Barone V., Mennucci B., Petersson G.A. (2009). G09a: Gaussian 09, Revision A.02.

