# Supplementary material for: Bioactive Diketopiperazines and Nucleoside Derivatives from a Sponge-Derived Streptomyces Species
Source: Mar Drugs. 2019 Oct 16;17(10):584. doi: 10.3390/md17100584 (PMC6835933; doi:10.3390/md17100584)
Supplement: Supplementary file 1 [file marinedrugs-17-00584-s001.pdf]

Supplementary Information

^{1}H NMR Spectrum of Compound **1** ($CDCl_3$)	S1
Expansion of the ^{1}H NMR Spectrum of Compound **1** ($CDCl_3$).	S2
^{13}C NMR Spectrum of Compound **1** ($CDCl_3$)	S3
^{1}H-^{1}H COSY NMR Spectrum of Compound **1** ($CDCl_3$)	S4
HSQC Spectrum of Compound **1** ($CDCl_3$)	S5
HMBC Spectrum of Compound **1** ($CDCl_3$)	S6
HRESIMS Spectrum of Compound **1**	S7
^{1}H NMR Spectrum of Compound **2** ($CDCl_3$)	S8
Expansion of ^{1}H NMR Spectrum of Compound **2** ($CDCl_3$)	S9
^{13}C NMR Spectrum of Compound **2** ($CDCl_3$)	S10
^{1}H-^{1}H COSY NMR Spectrum of Compound **2** ($CDCl_3$)	S11
HSQC Spectrum of Compound **2** ($CDCl_3$)	S12
HMBC Spectrum of Compound **2** ($CDCl_3$)	S13
HRESIMS Spectrum of Compound **2**	S14
^{1}H NMR Spectrum and Expansion of ^{1}H NMR of Compound **3** (DMSO-d_6)	S15
^{13}C NMR Spectrum of Compound **3** (DMSO-d_6)	S16
^{1}H-^{1}H COSY NMR Spectrum of Compound **3** (DMSO-d_6)	S17
HSQC Spectrum of Compound **3** (DMSO-d_6)	S18
HMBC Spectrum of Compound **3** (DMSO-d_6)	S19
ROESY Spectrum of Compound **3** (DMSO-d_6)	S20
HRESIMS Spectrum of Compound **3**	S21
^{1}H NMR Spectrum of Compound **4** (DMSO-d_6)	S22
Expansion of ^{1}H NMR Spectrum of Compound **4** (DMSO-d_6)	S23
^{13}C NMR Spectrum of Compound **4** (DMSO-d_6)	S24
^{1}H-^{1}H COSY NMR Spectrum of Compound **4** (DMSO-d_6)	S25
HSQC Spectrum of Compound **4** (DMSO-d_6)	S26
HMBC Spectrum of Compound **4** (DMSO-d_6)	S27
HRESIMS Spectrum of Compound **4**	S28
^{1}H NMR Spectrum of Compound **5** ($CDCl_3$)	S29
^{13}C NMR Spectrum of Compound **5** ($CDCl_3$)	S30
^{1}H-^{1}H COSY NMR Spectrum of Compound **5** ($CDCl_3$)	S31
HSQC Spectrum of Compound **5** ($CDCl_3$)	S32
HMBC Spectrum of Compound **5** ($CDCl_3$)	S33
^{1}H NMR Spectrum of Compound **6** ($CDCl_3$)	S34
^{13}C NMR Spectrum of Compound **6** ($CDCl_3$)	S35
^{1}H-^{1}H COSY NMR Spectrum of Compound **6** ($CDCl_3$)	S36
HSQC Spectrum of Compound **6** ($CDCl_3$)	S37
HMBC Spectrum of Compound **6** ($CDCl_3$)	S38
HPLC trace for Marfey's-derivatized hydrolysates of compound **1**, **2**, **5**, **6** and derivatized standard amino acids	S39
Concentration-response profiles for compounds **1-6**	S40

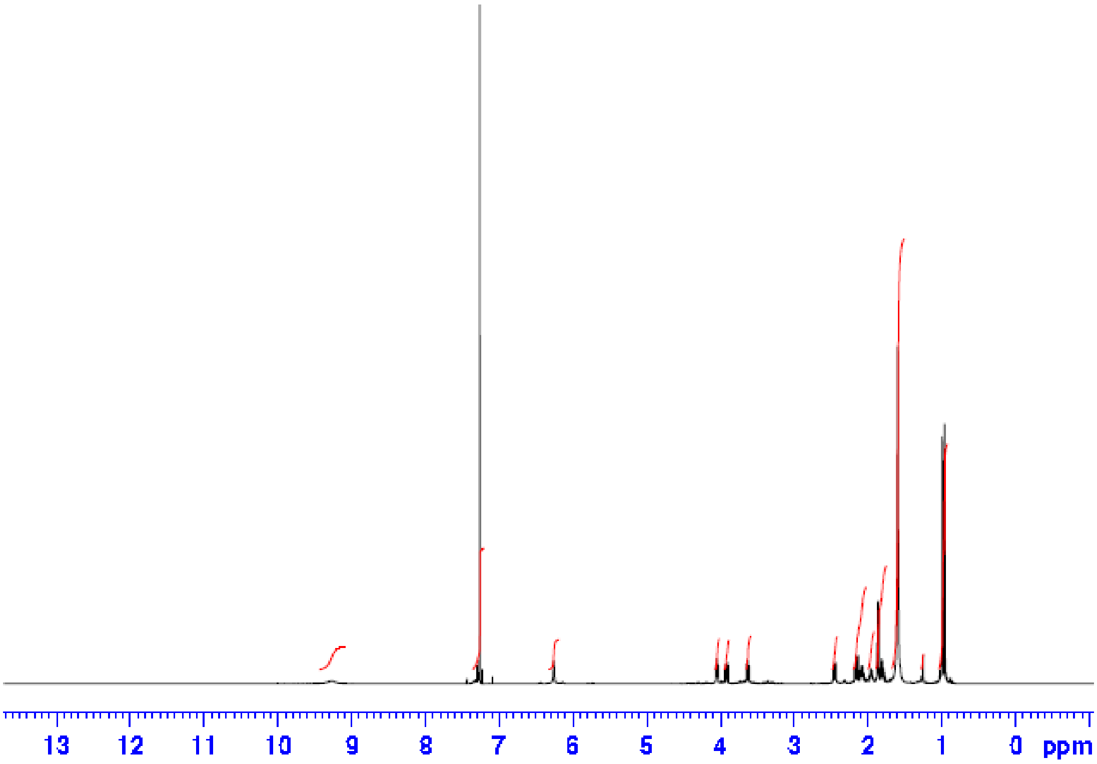

^{1}H NMR Spectrum of Compound **1** ($CDCl_3$).

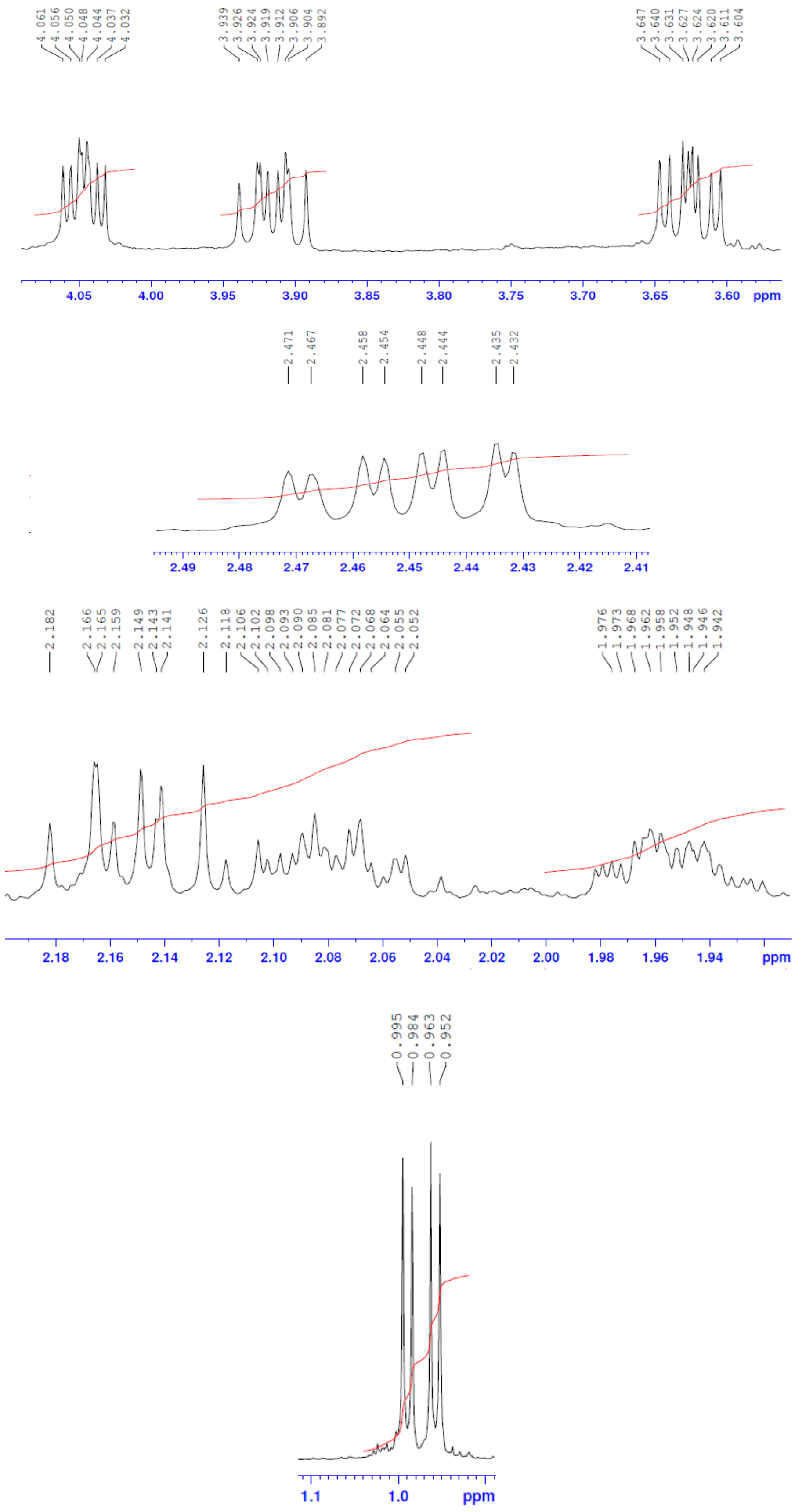

Expansion of the ^{1}H NMR Spectrum of Compound **1** ($CDCl_3$).

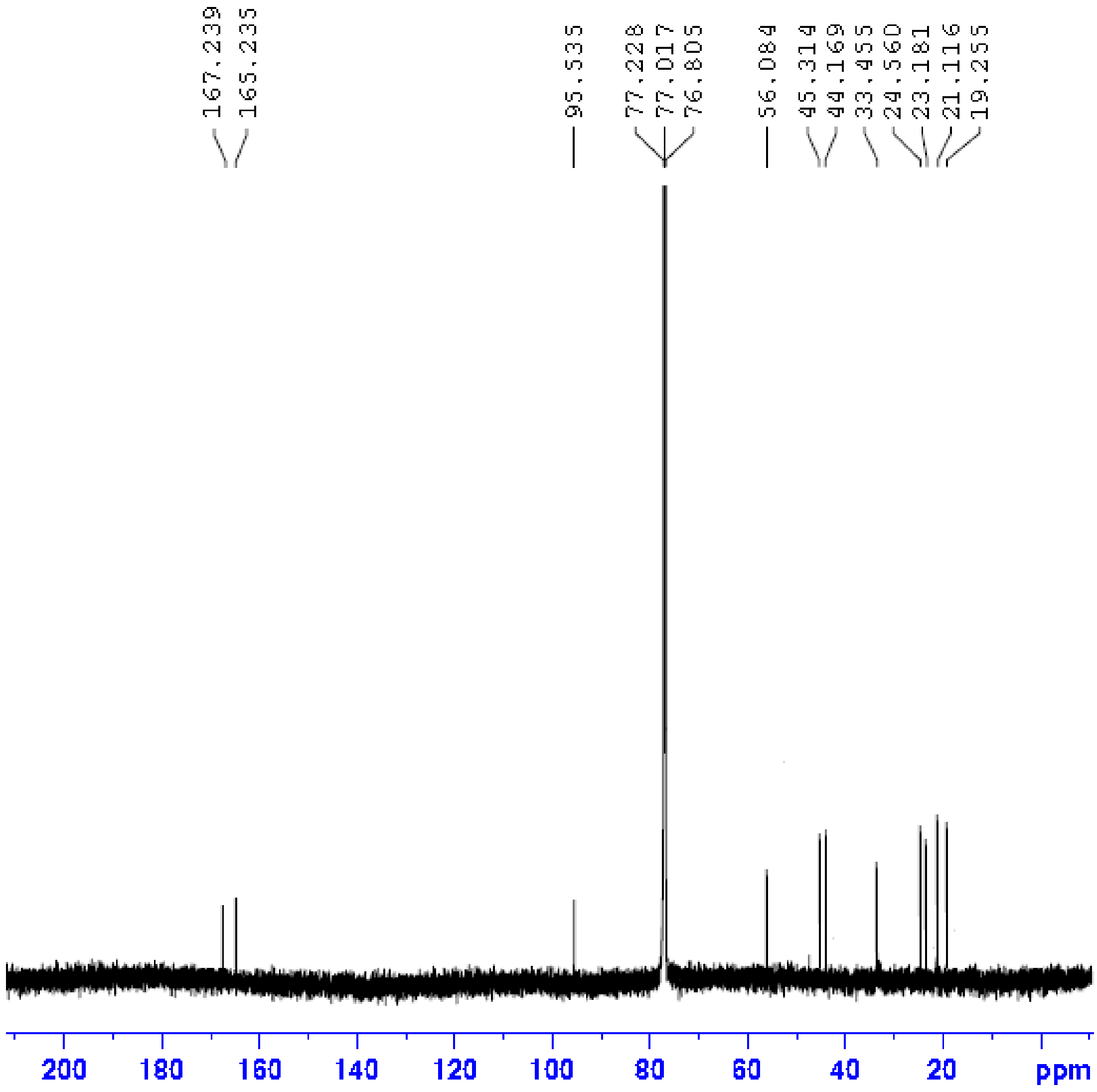

^{13}C NMR Spectrum of Compound **1** ($CDCl_3$).

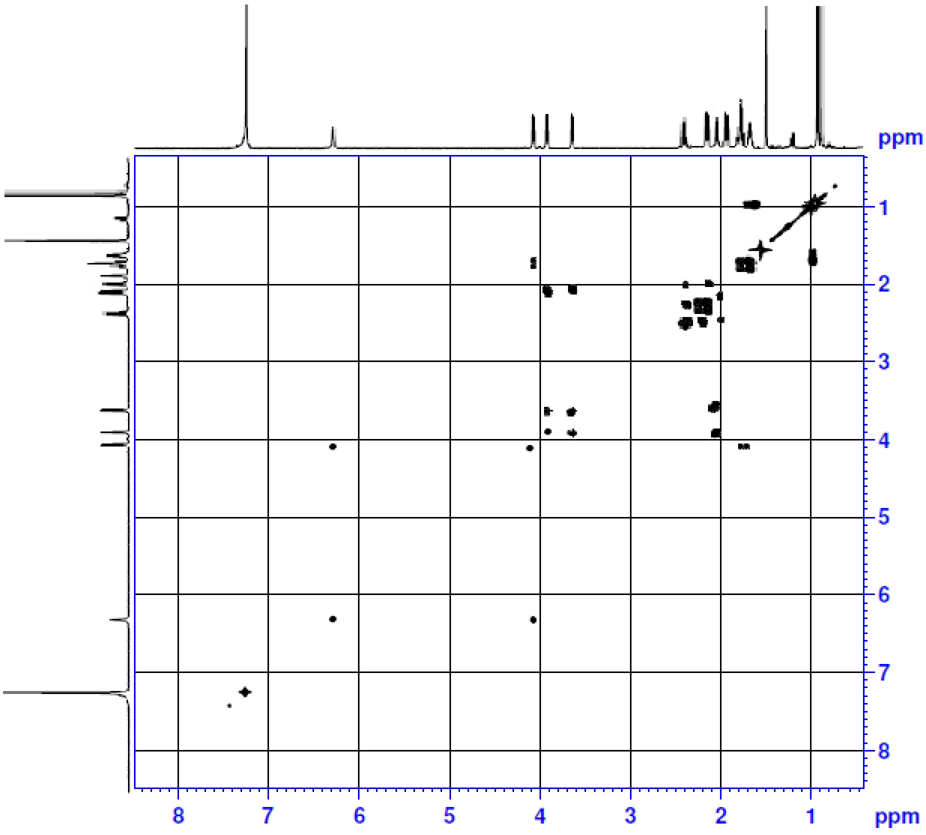

^1H-^1H COSY NMR Spectrum of Compound **1** ($CDCl_3$).

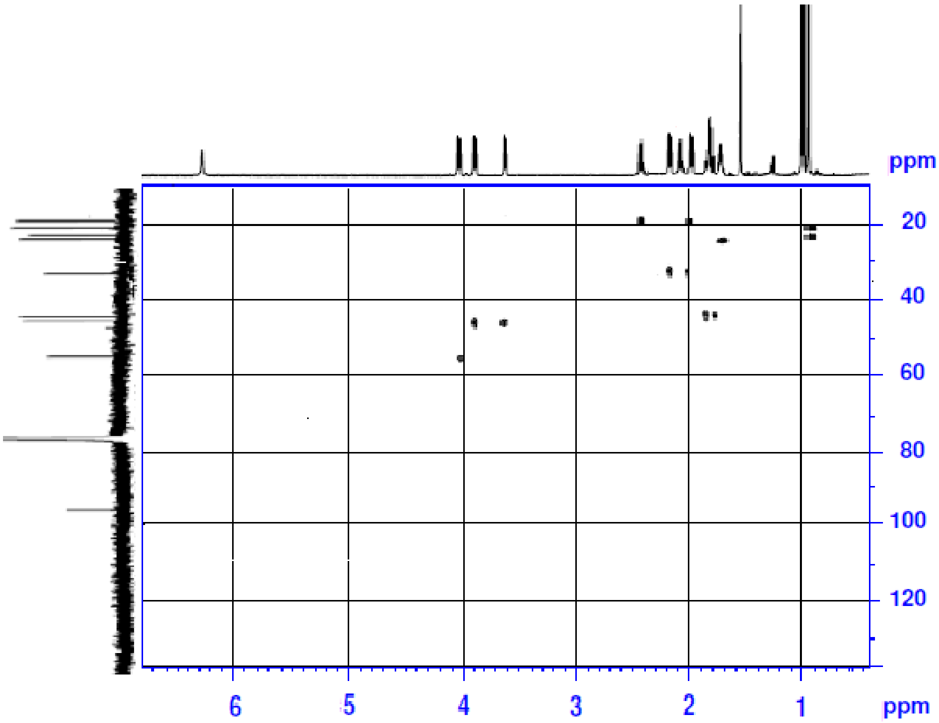

HSQC Spectrum of Compound **1** ($CDCl_3$)

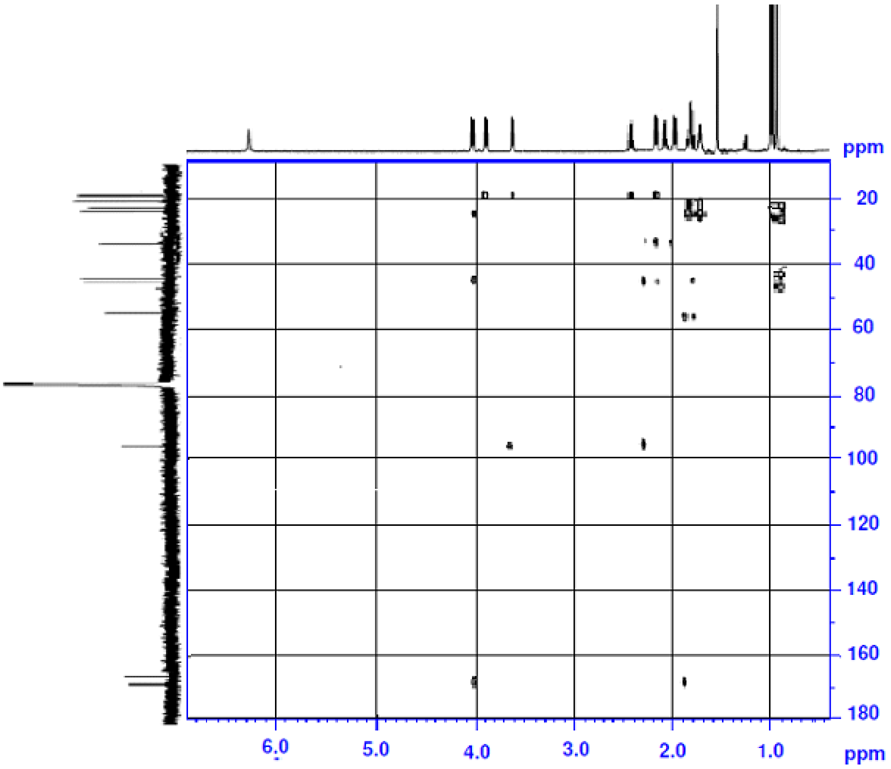

HMBC Spectrum of Compound **1** ($CDCl_3$)

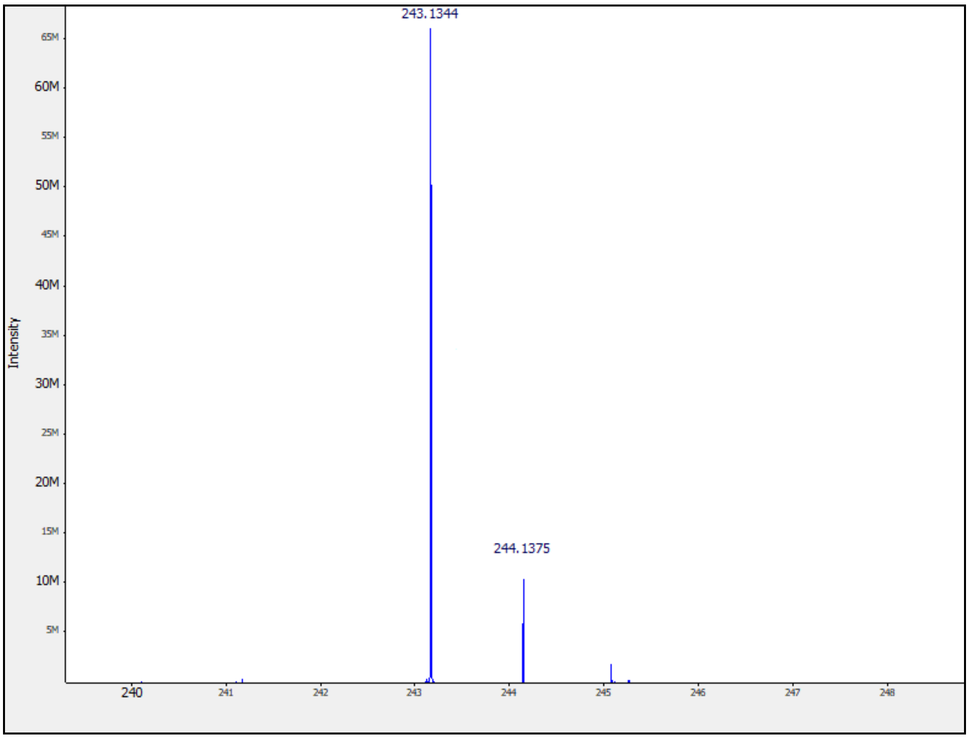

HRESIMS of Compound **1.**

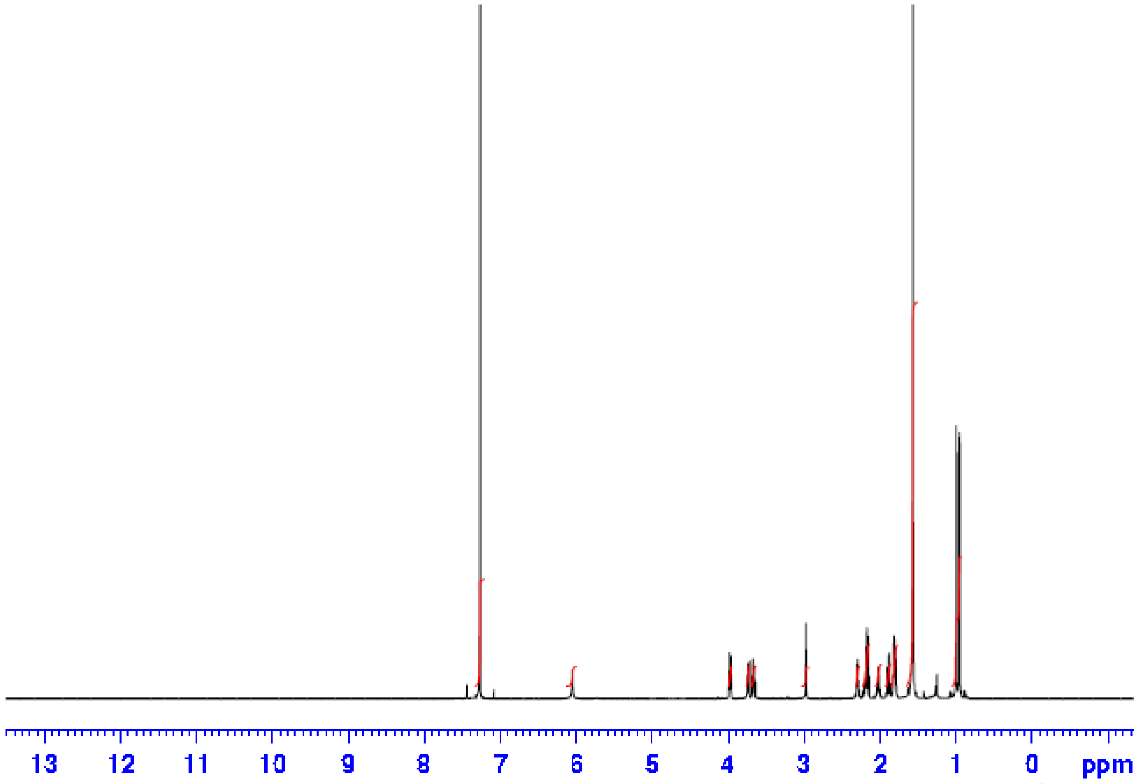

^{1}H NMR Spectrum of Compound **2** (850 MHz, $CDCl_3$).

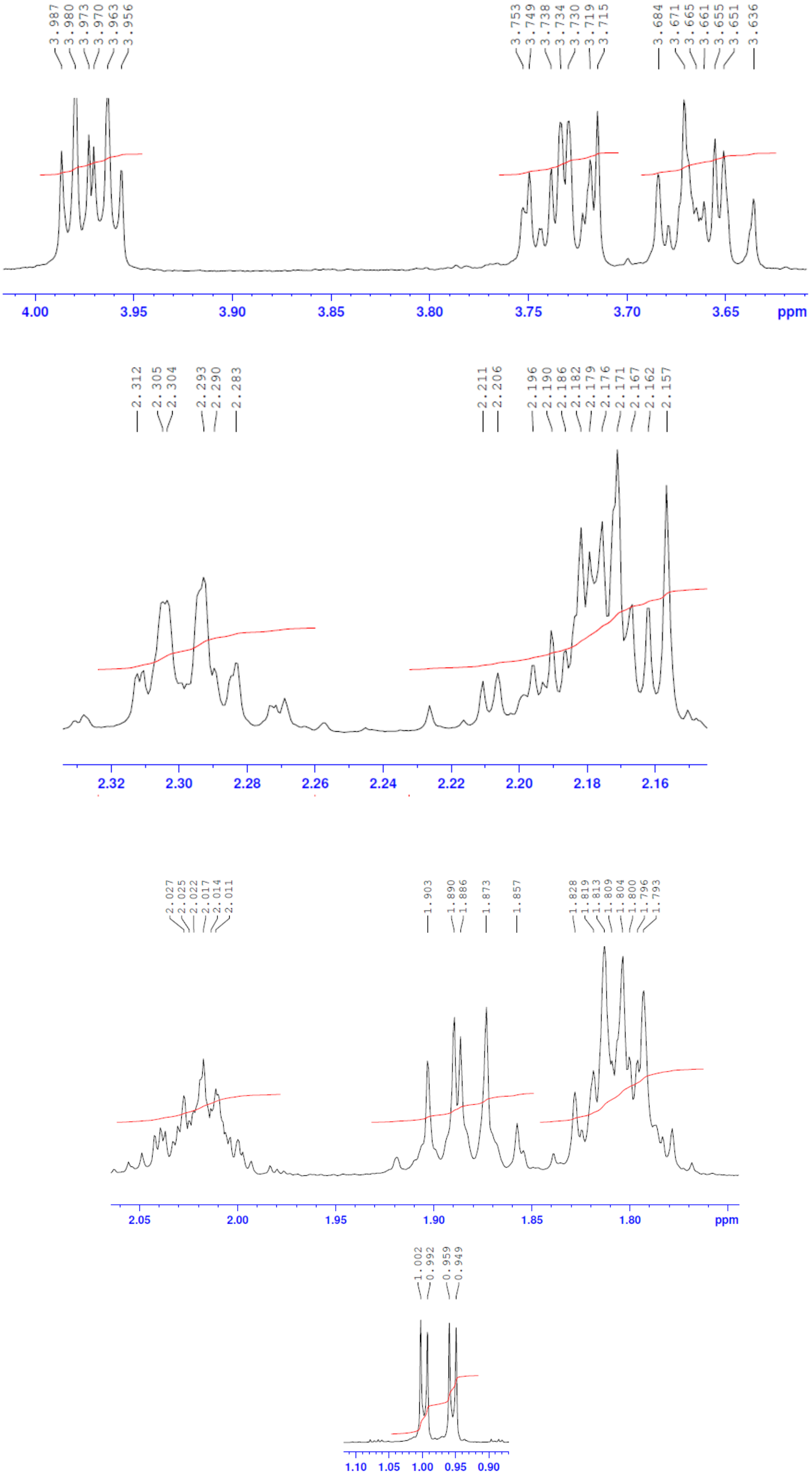

Expansion of ^{1}H NMR Spectrum of Compound **2** (850 MHz, $CDCl_3$).

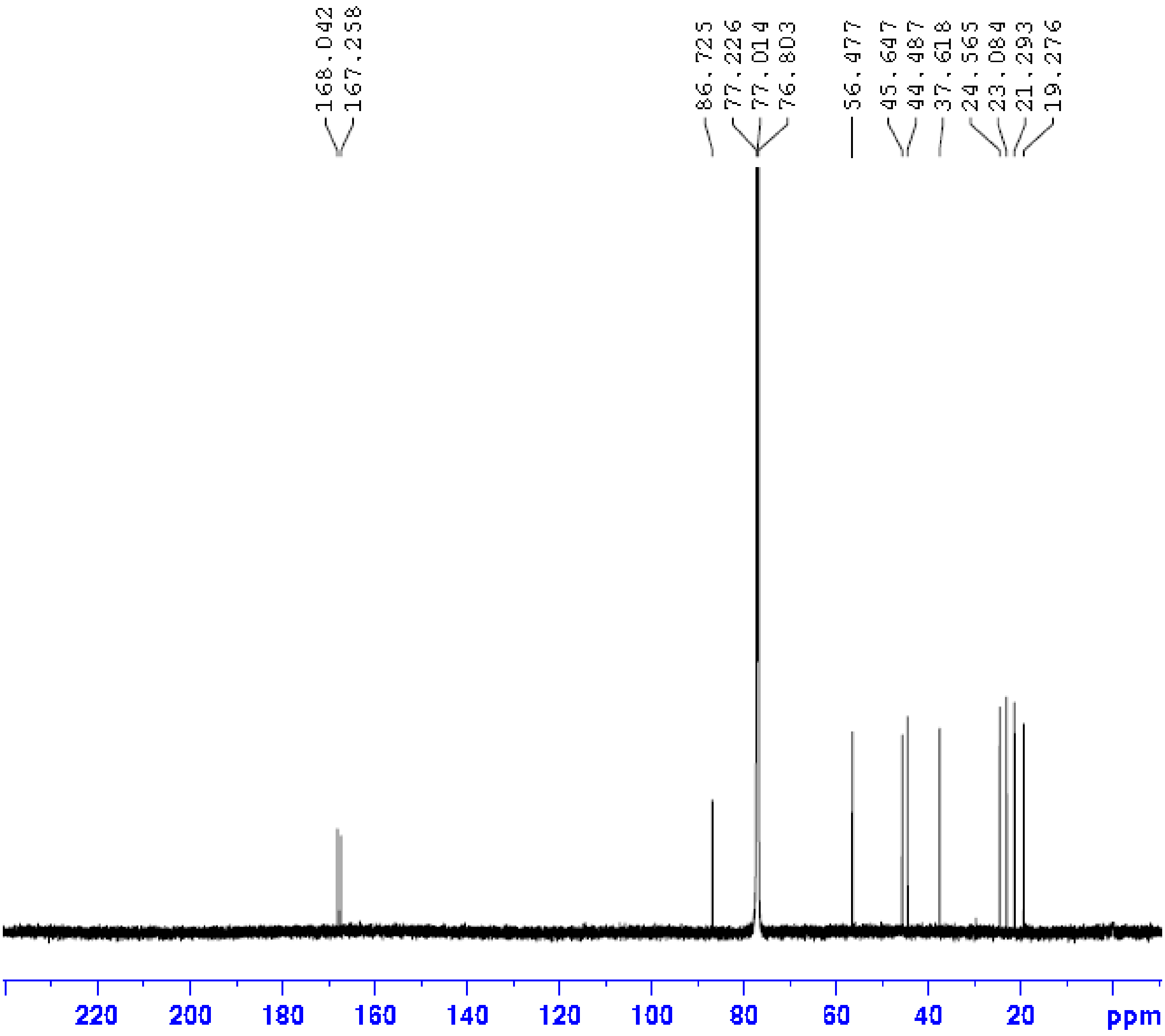

^{13}C NMR Spectrum of Compound **2** (213 MHz, $CDCl_3$).

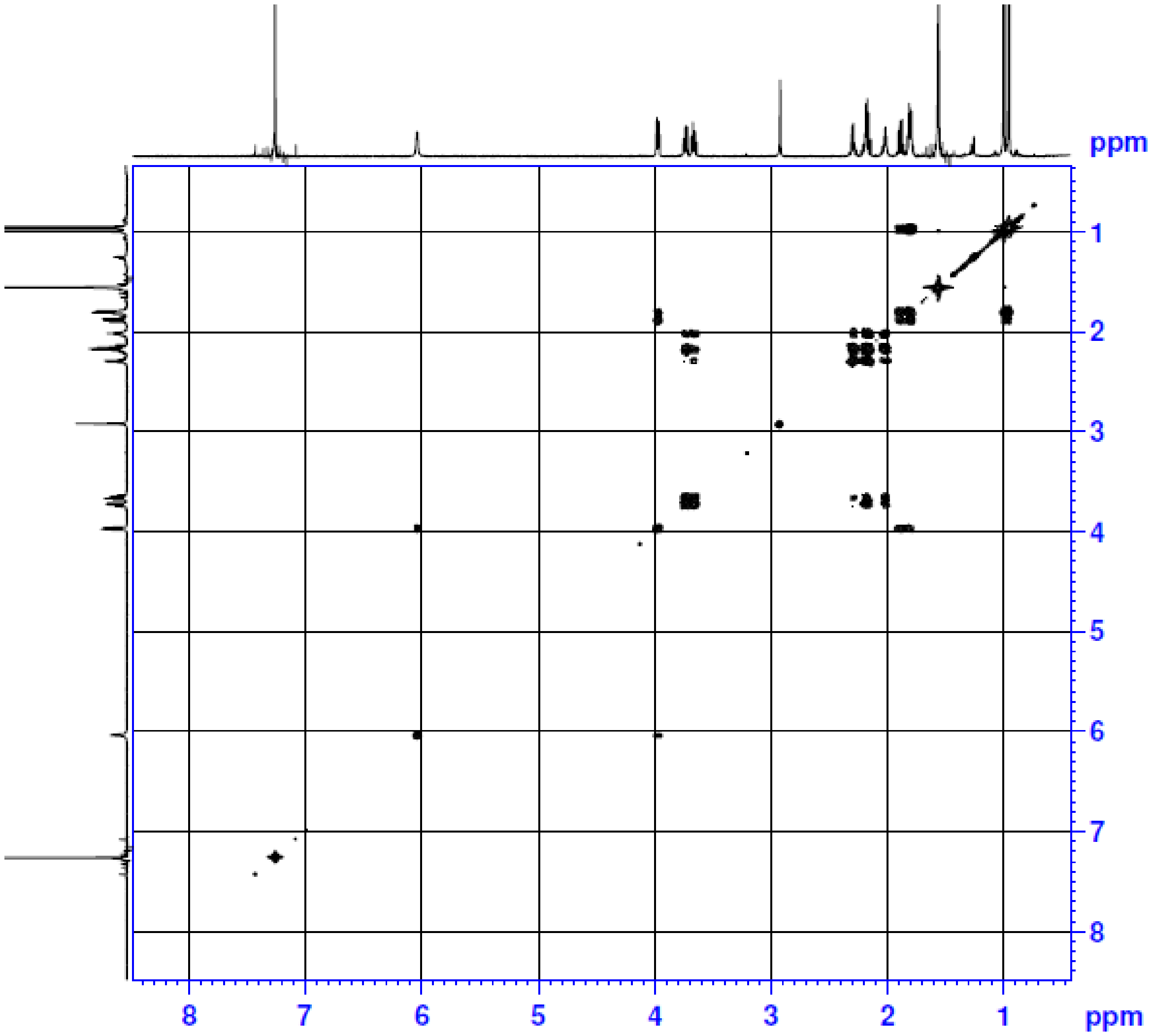

^{1}H-^{1}H COSY NMR Spectrum of Compound **2** (850 MHz, $CDCl_3$).

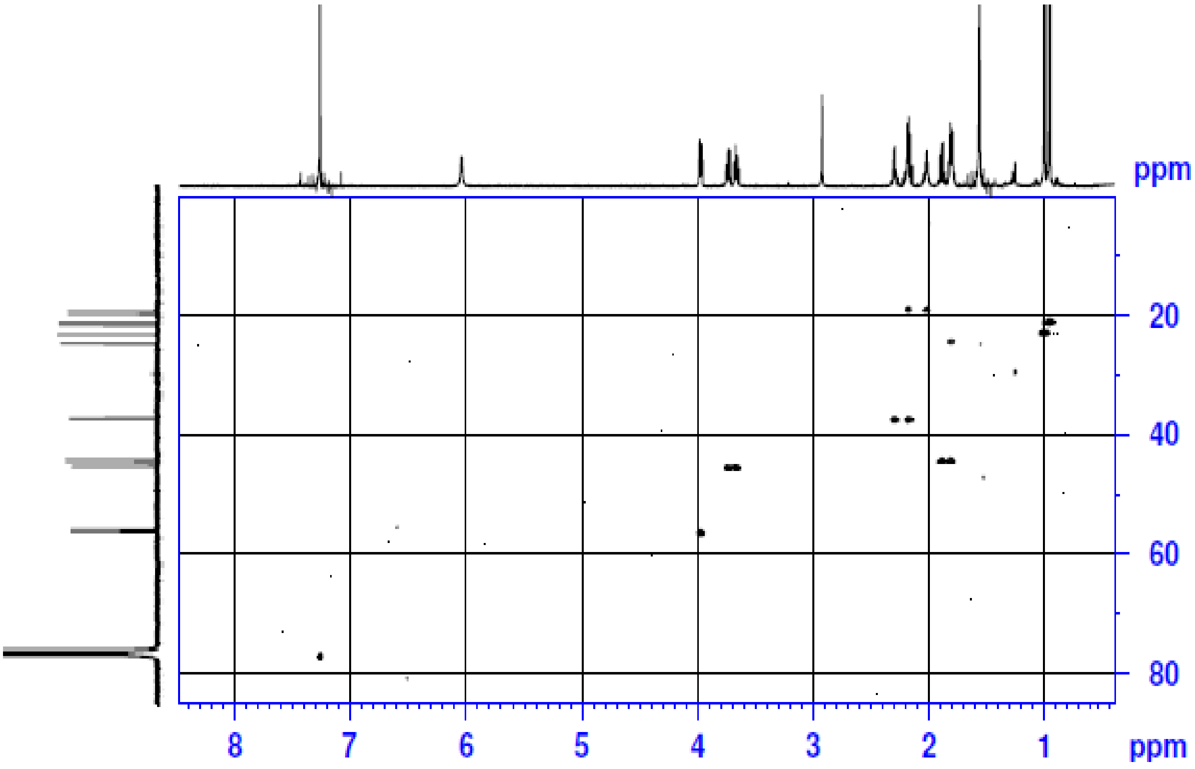

HSQC Spectrum of Compound **2** ($CDCl_3$)

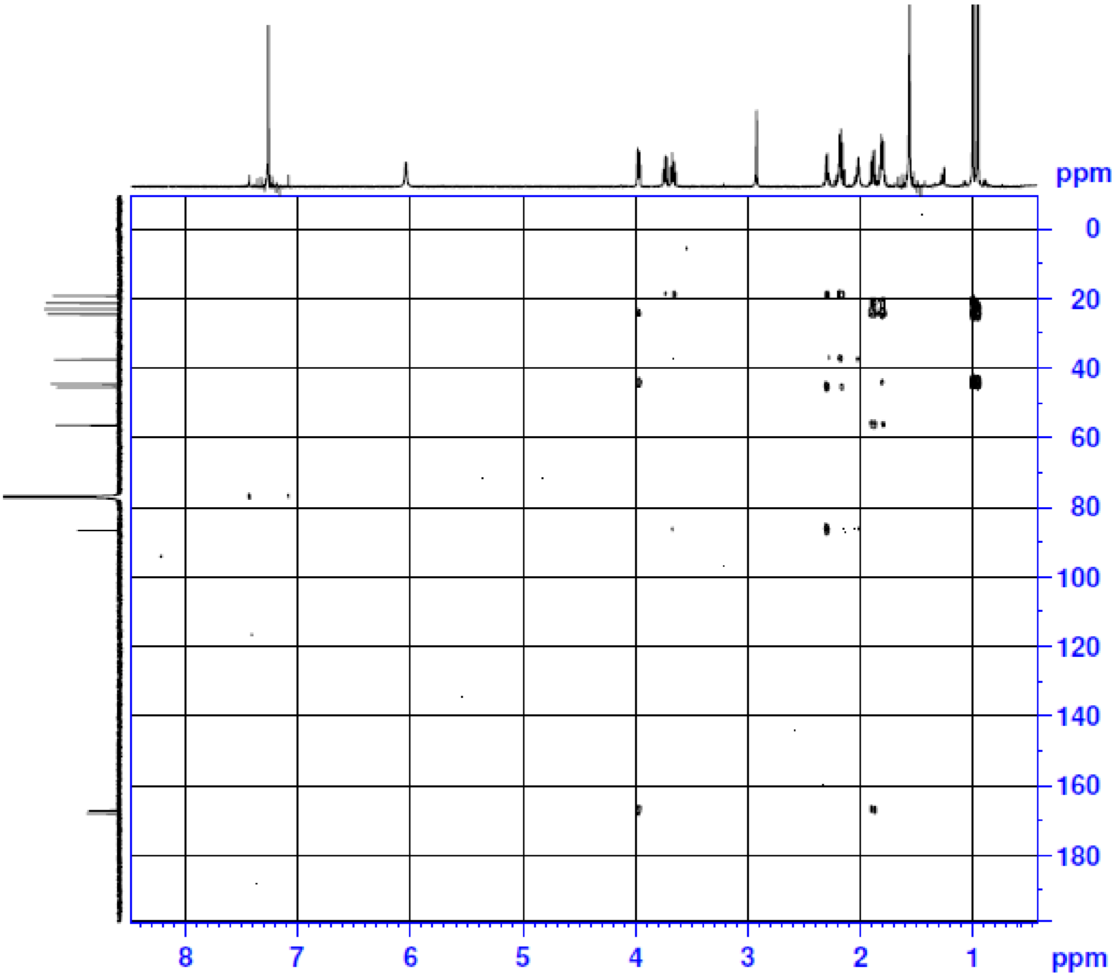

HMBC Spectrum of Compound **2** (850 MHz, $CDCl_3$)

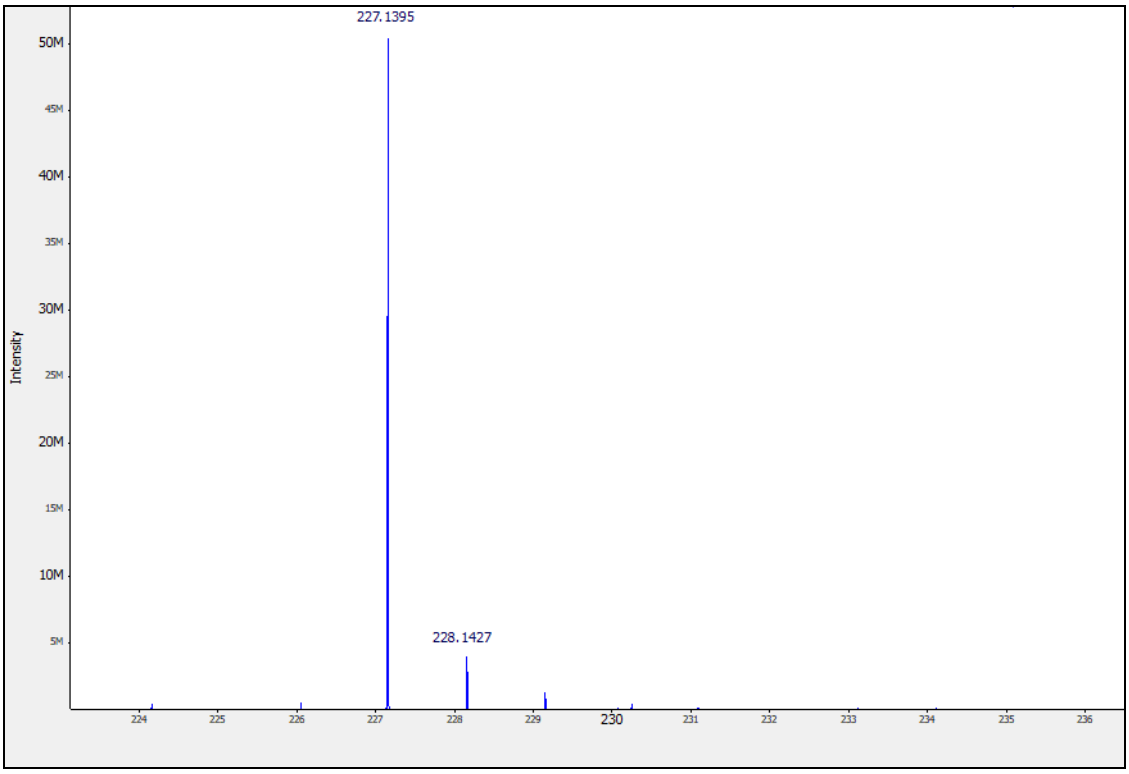

HRESIMS Spectrum of Compound **2**.

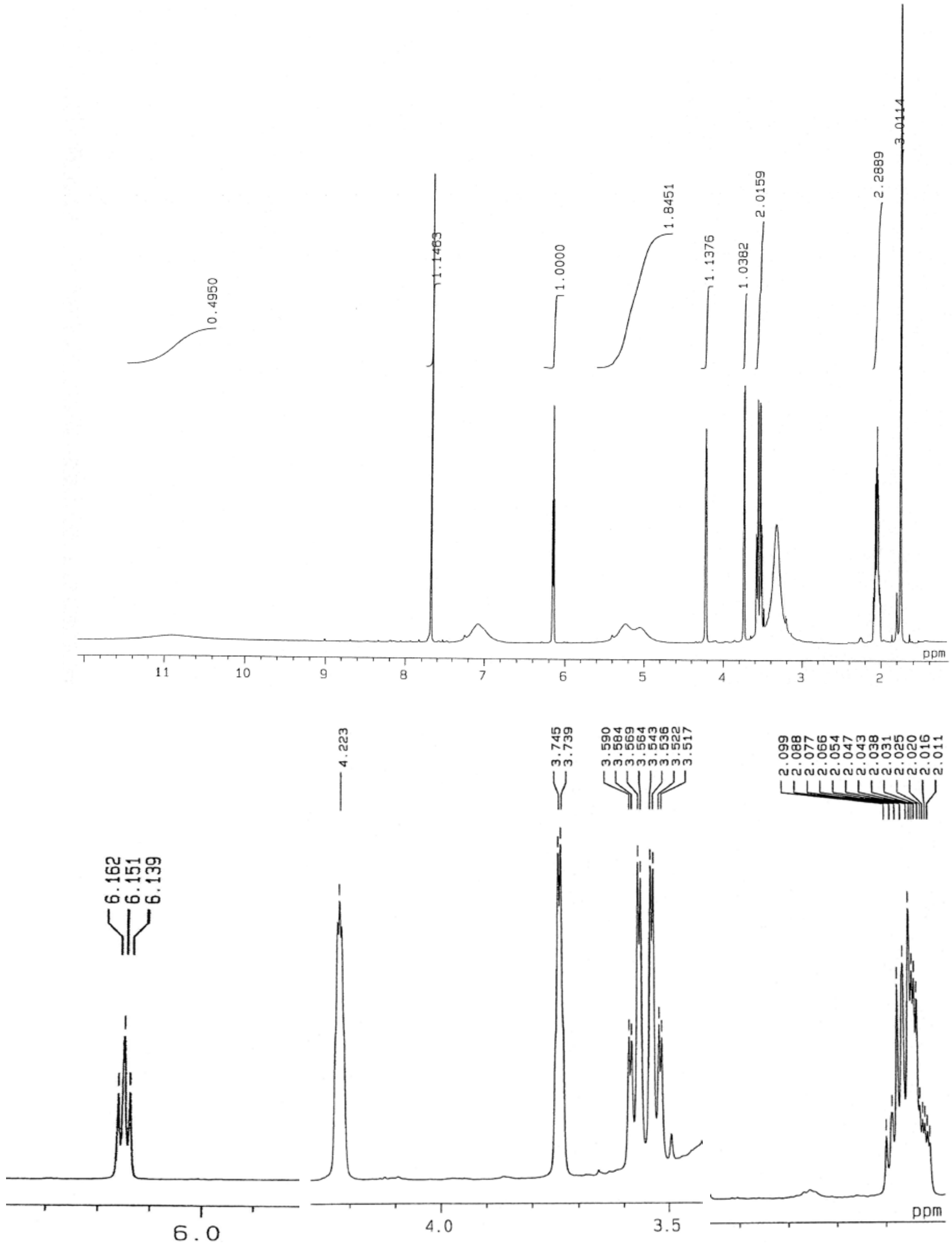

^{1}H NMR Spectrum and Expansion of ^{1}H NMR of Compound **3** (600 MHz, DMSO-d_6).

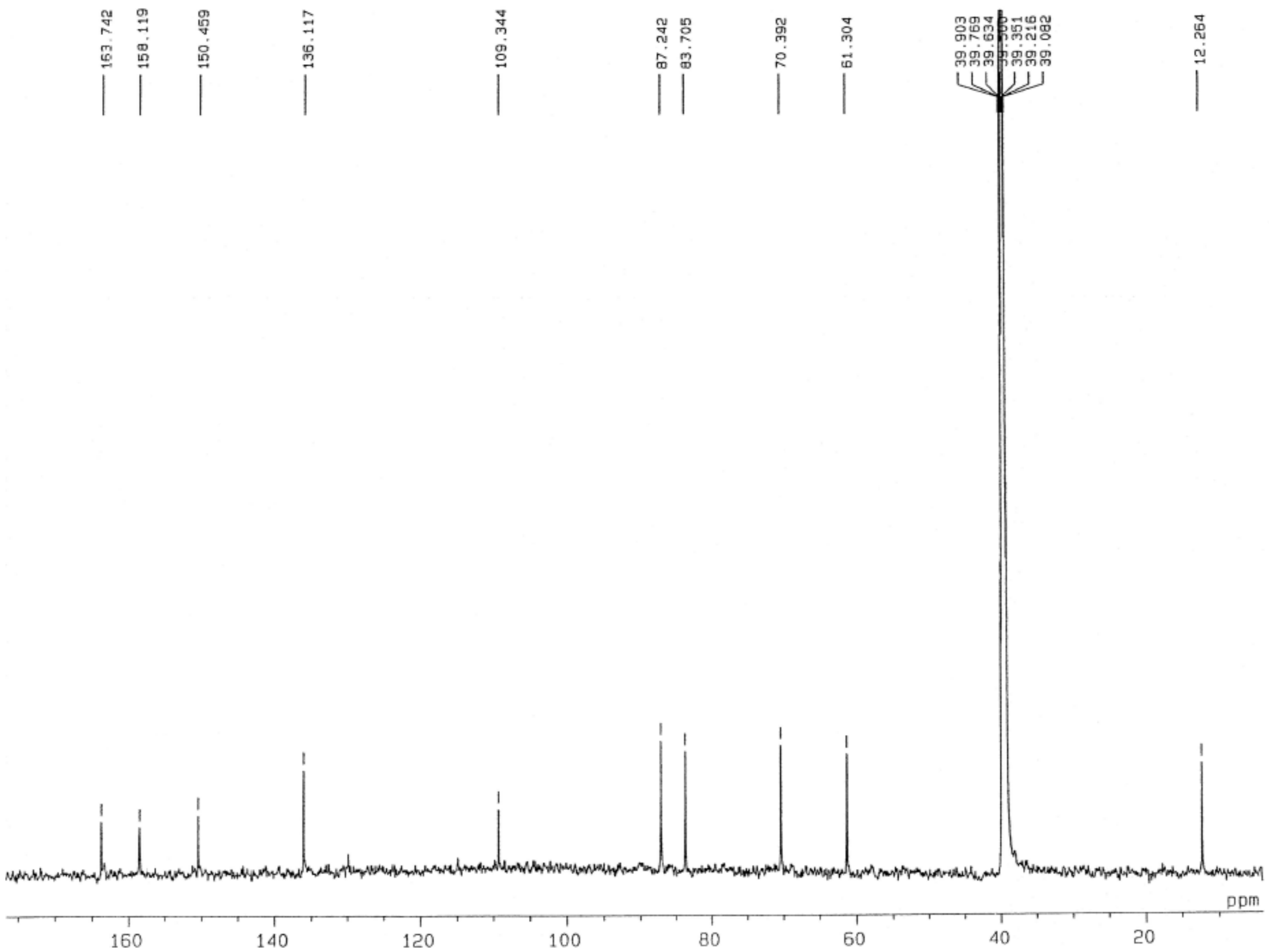

^{13}C NMR Spectrum of Compound **3** (600 MHz, DMSO-d_6).

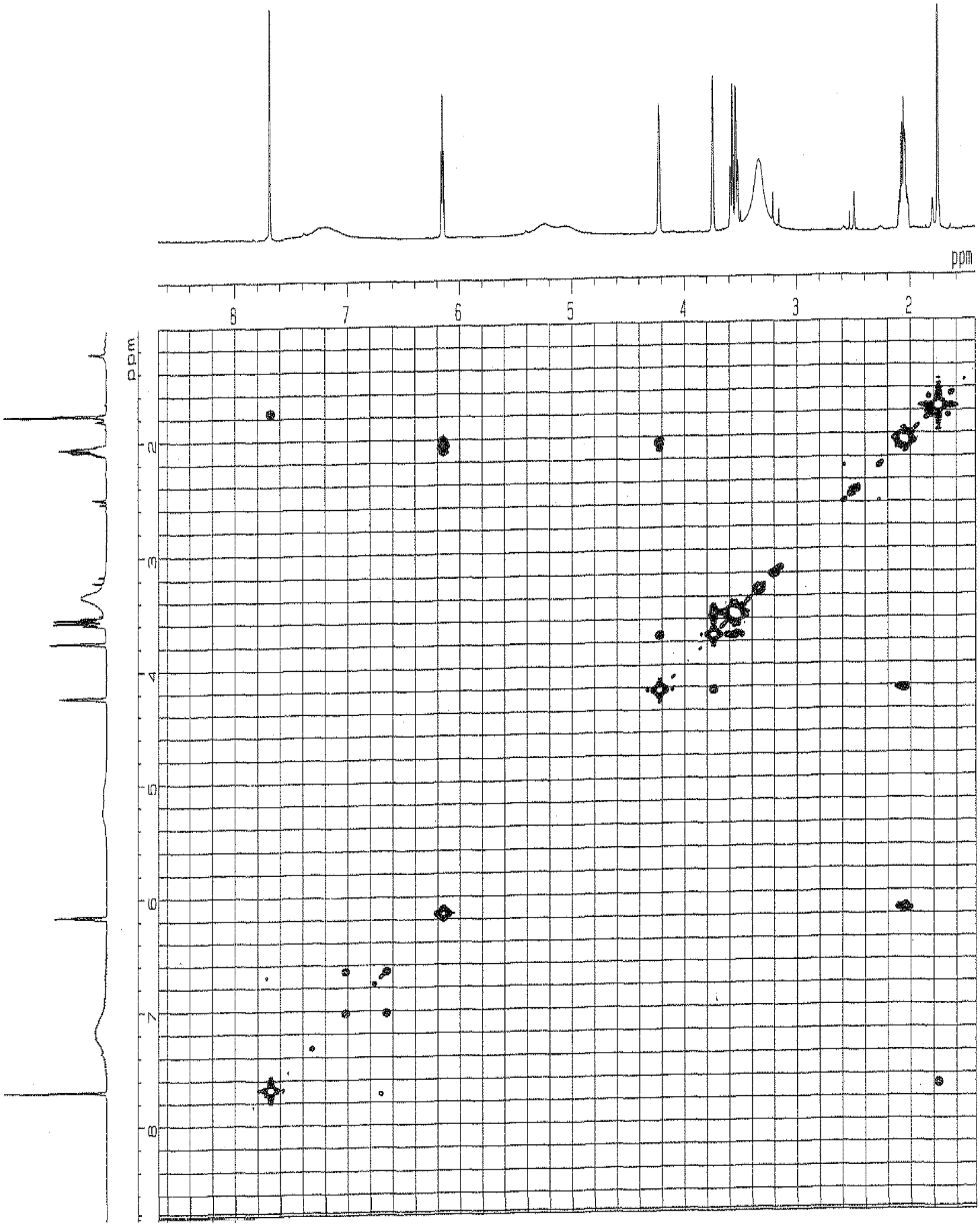

^{1}H-^{1}H COSY Spectrum of Compound **3** (600 MHz, DMSO-d_6)

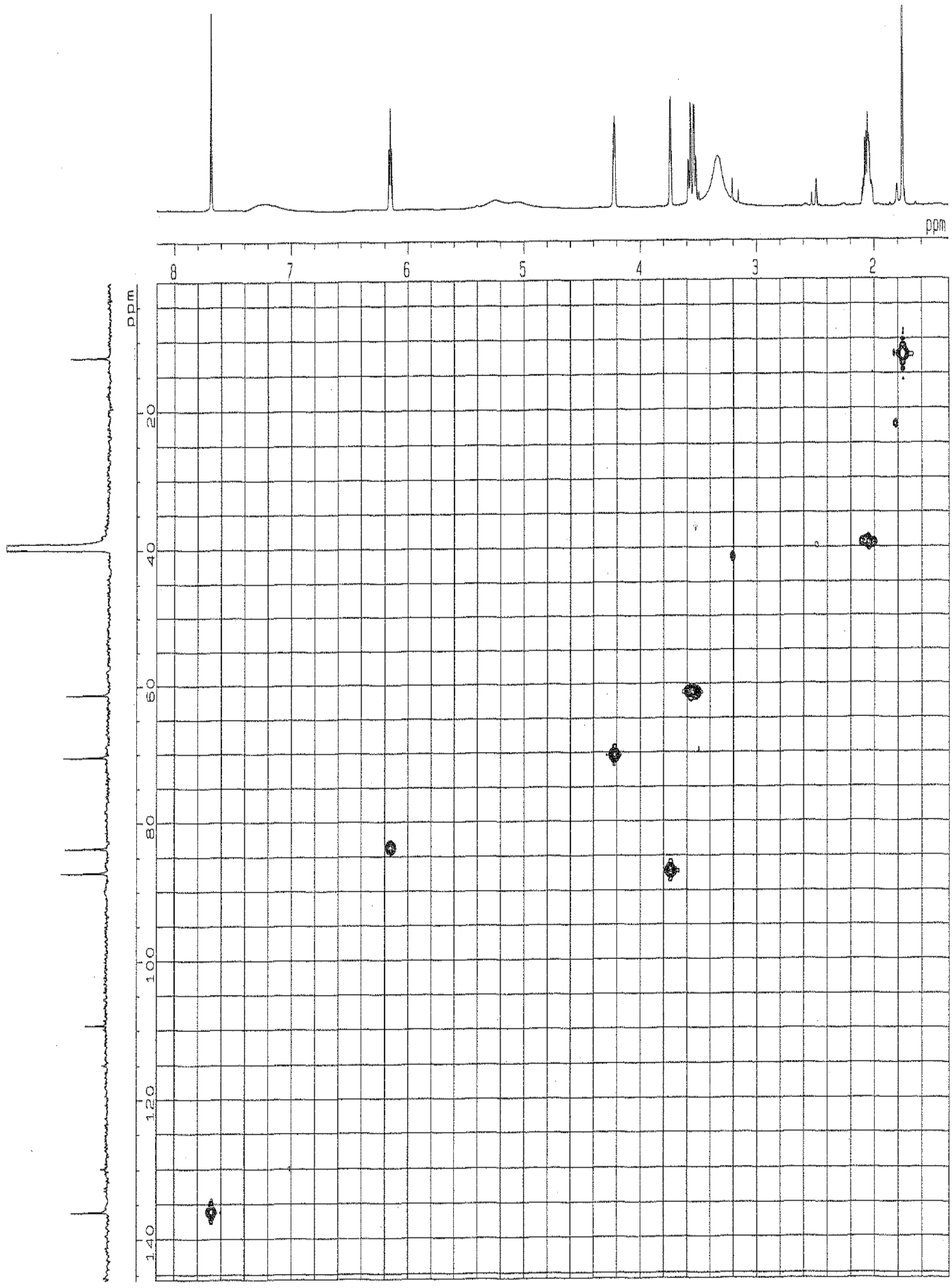

HSQC Spectrum of Compound **3** (DMSO-d_6)

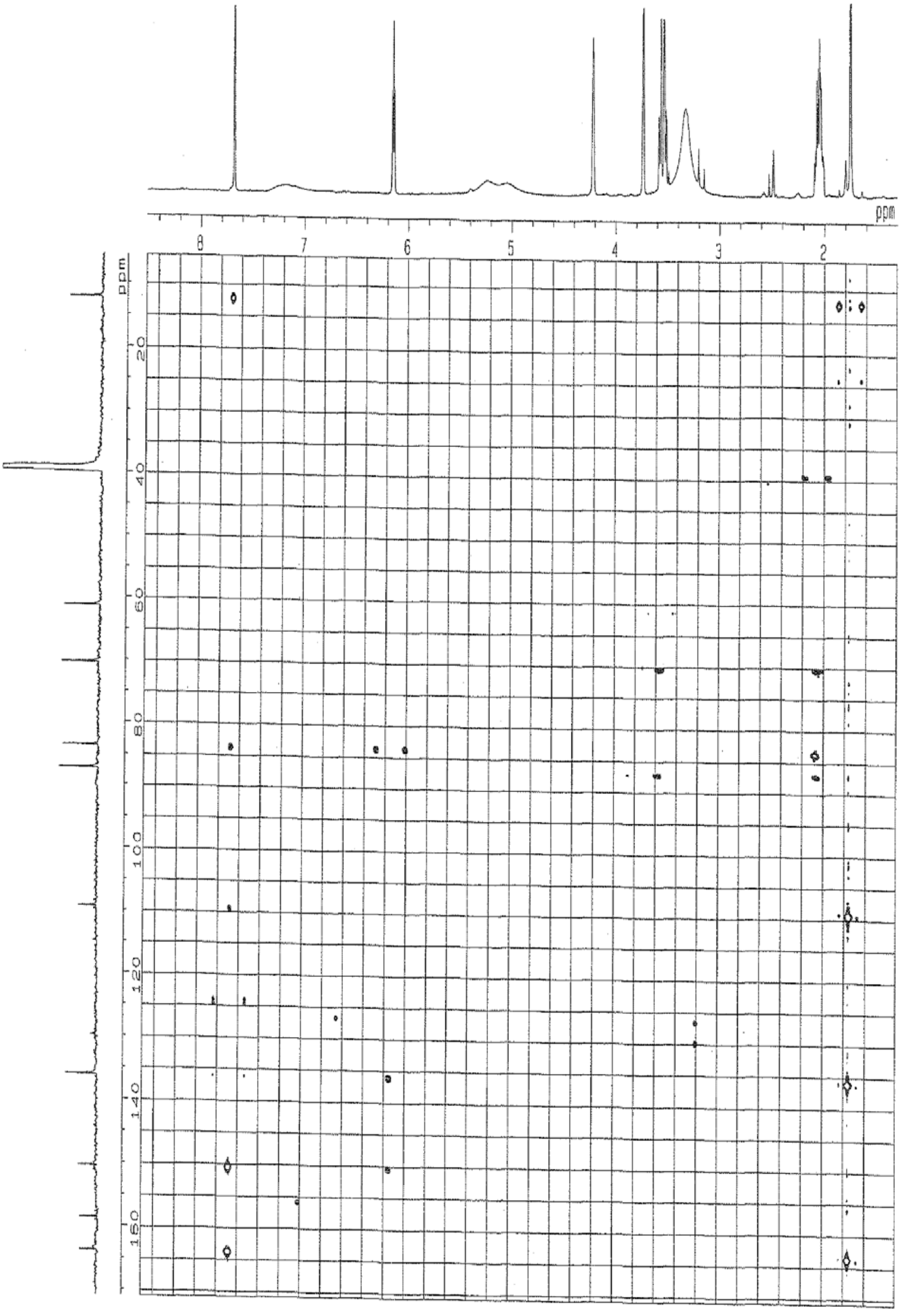

HMBC Spectrum of Compound **3** (DMSO-d_6)

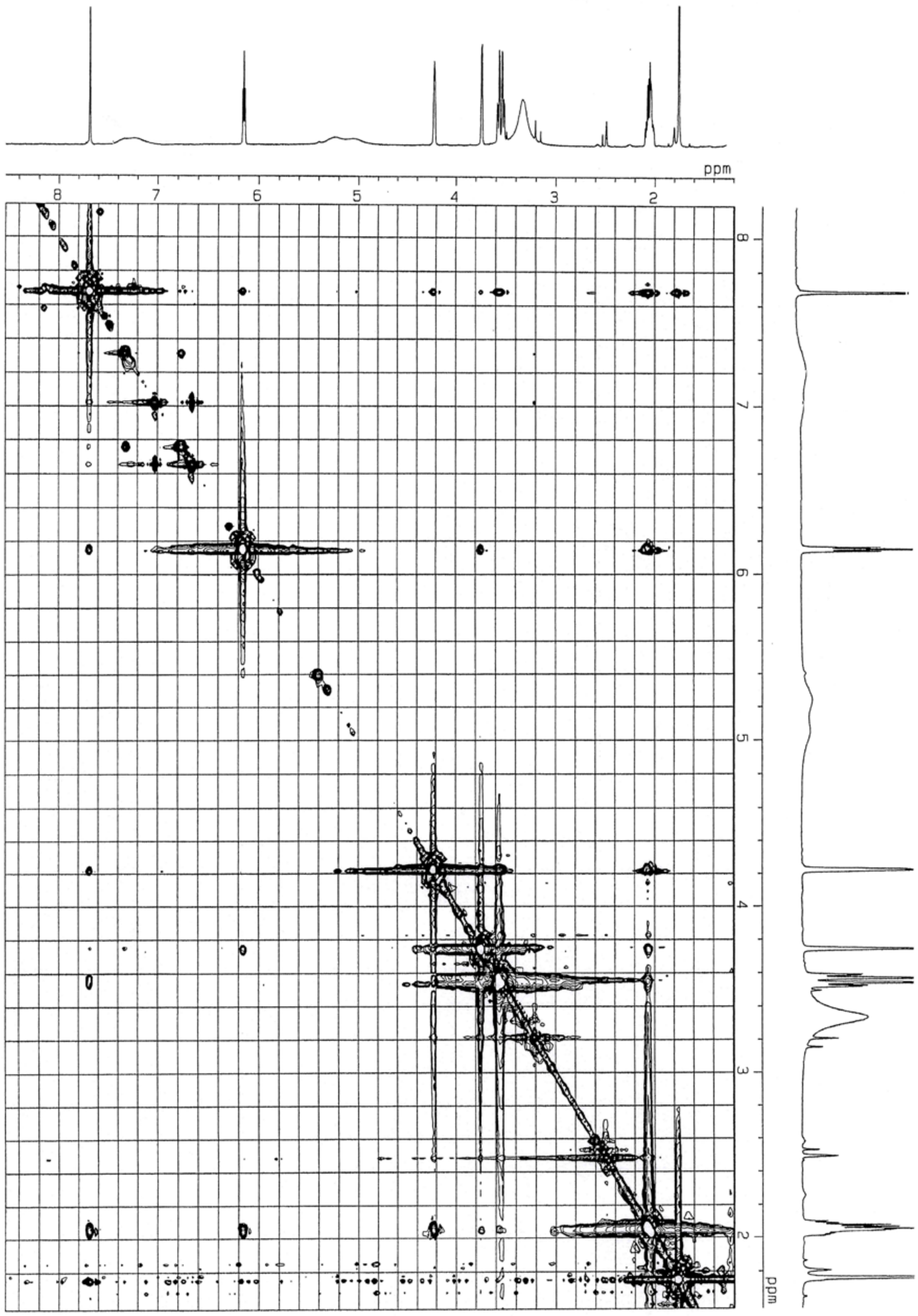

ROESY Spectrum of Compound **3** (DMSO-d_6)

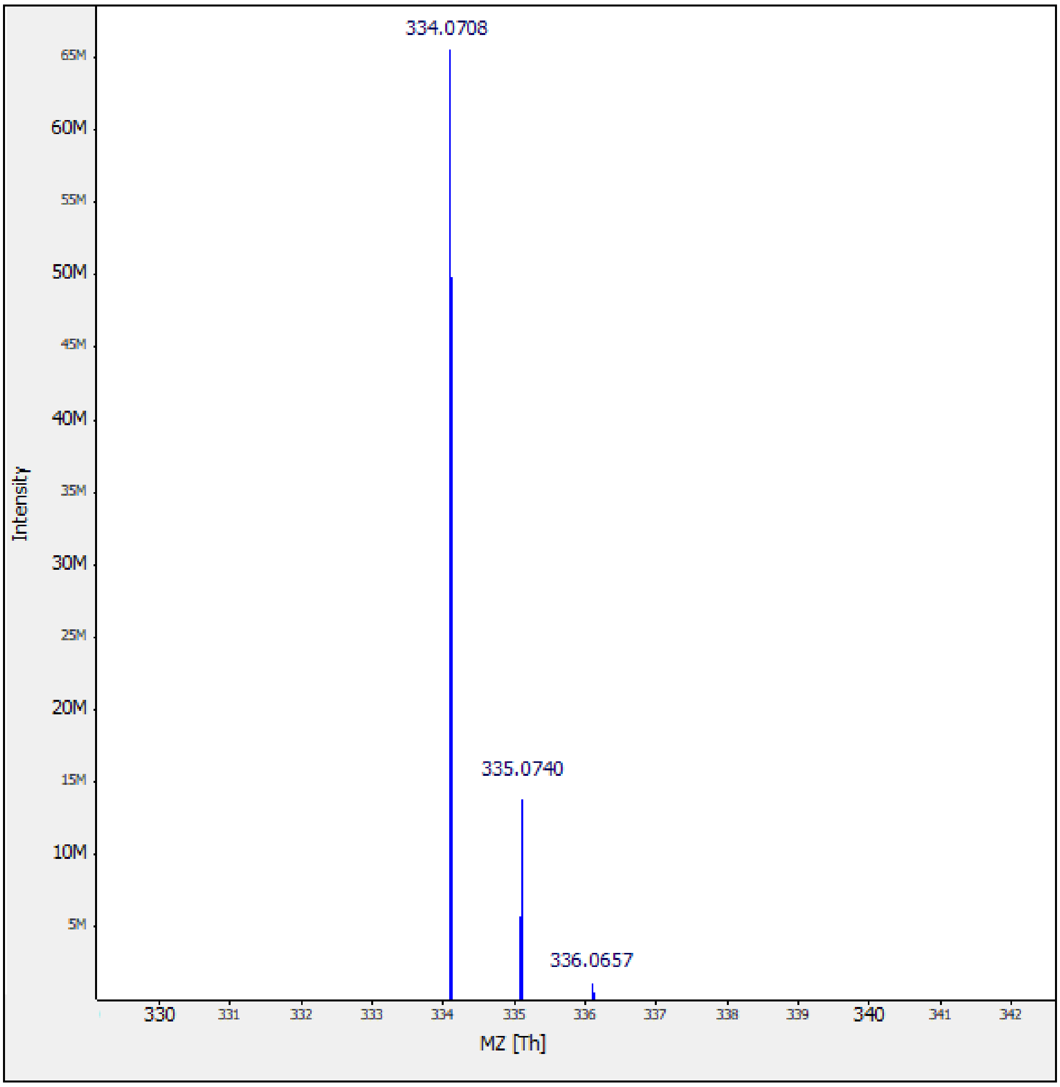

HRESIMS Spectrum of Compound **3**.

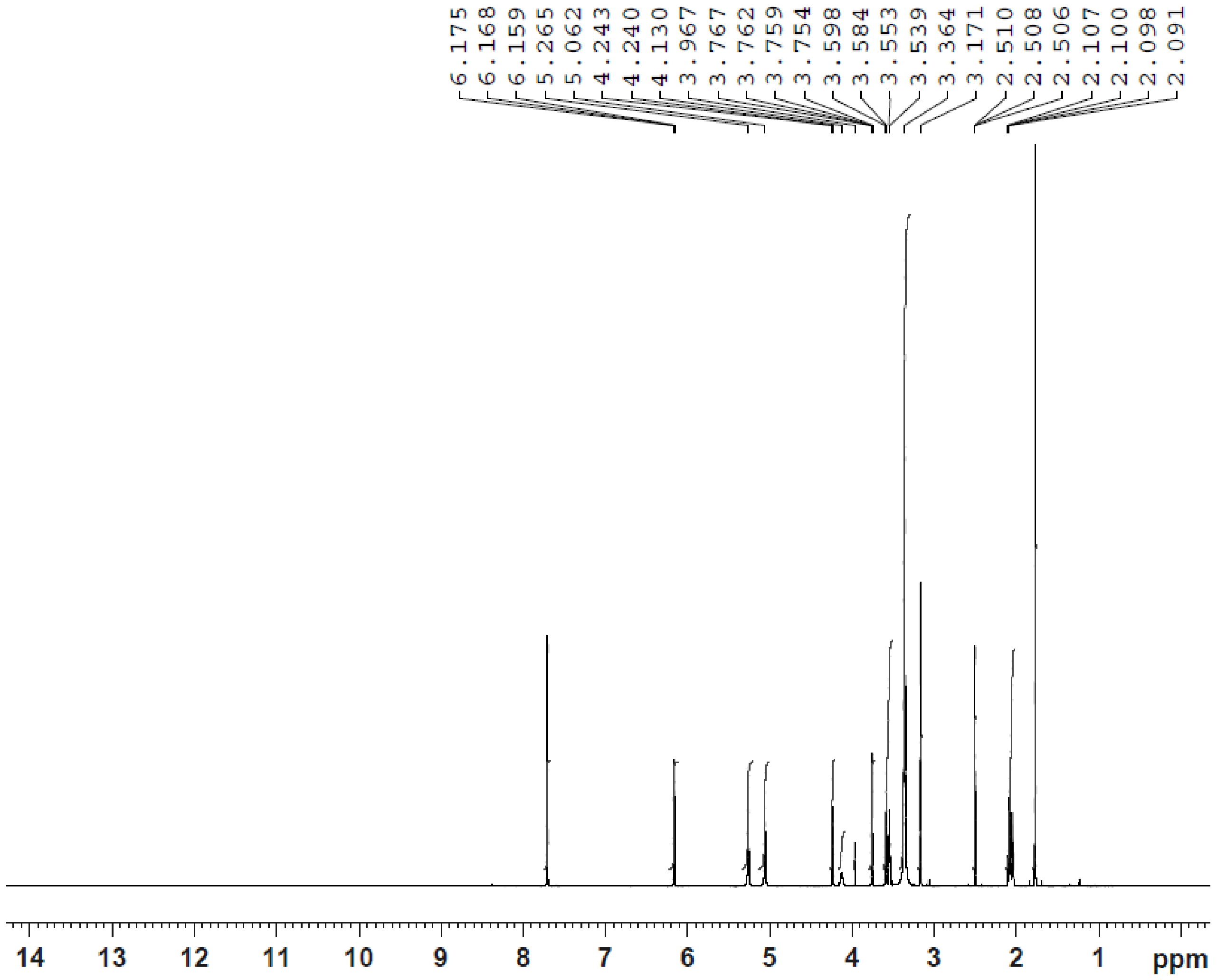

^{1}H NMR Spectrum of Compound **4** (DMSO-d_6)

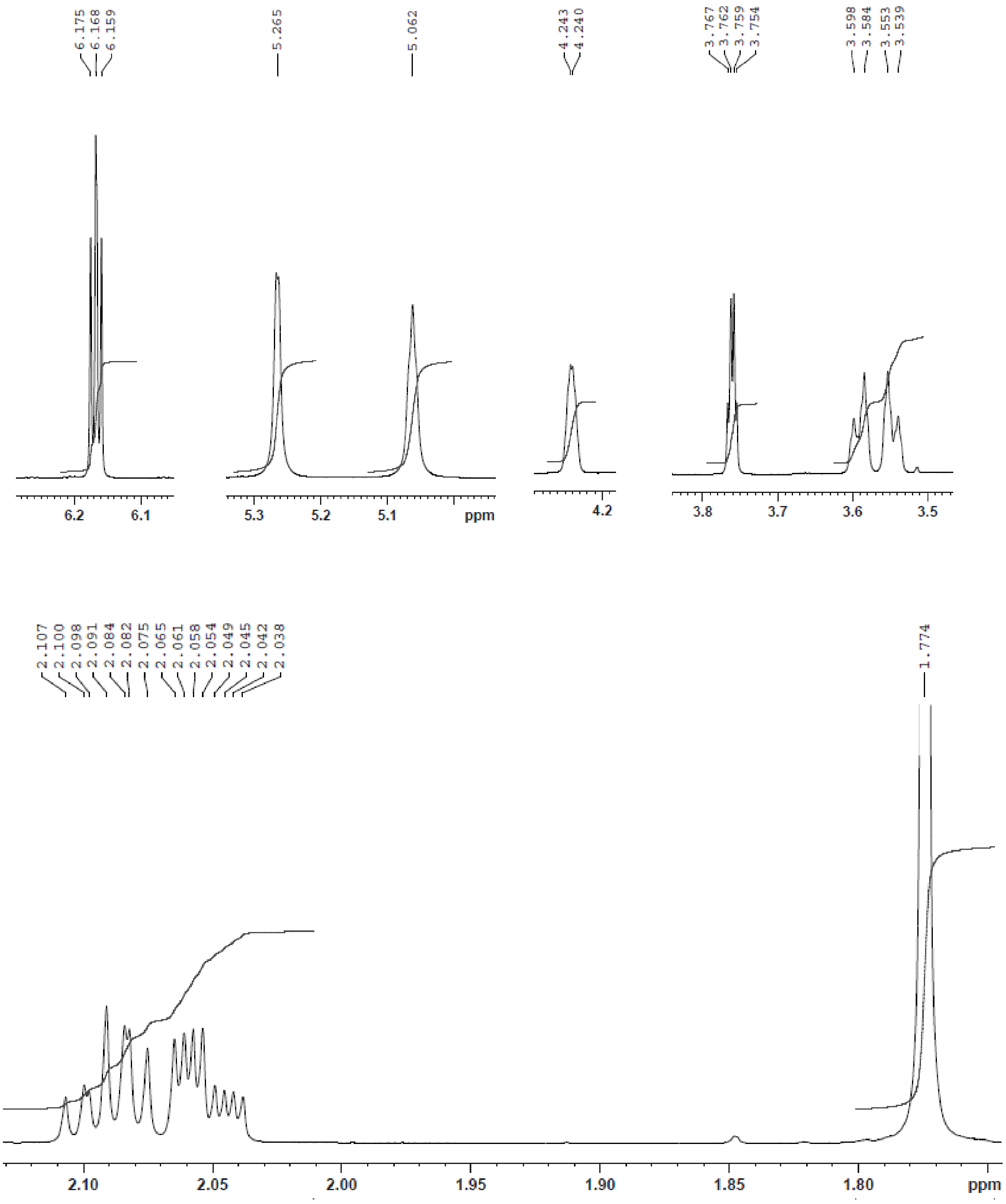

Expansion of the NMR Spectrum of Compound **4** (DMSO-d_6)

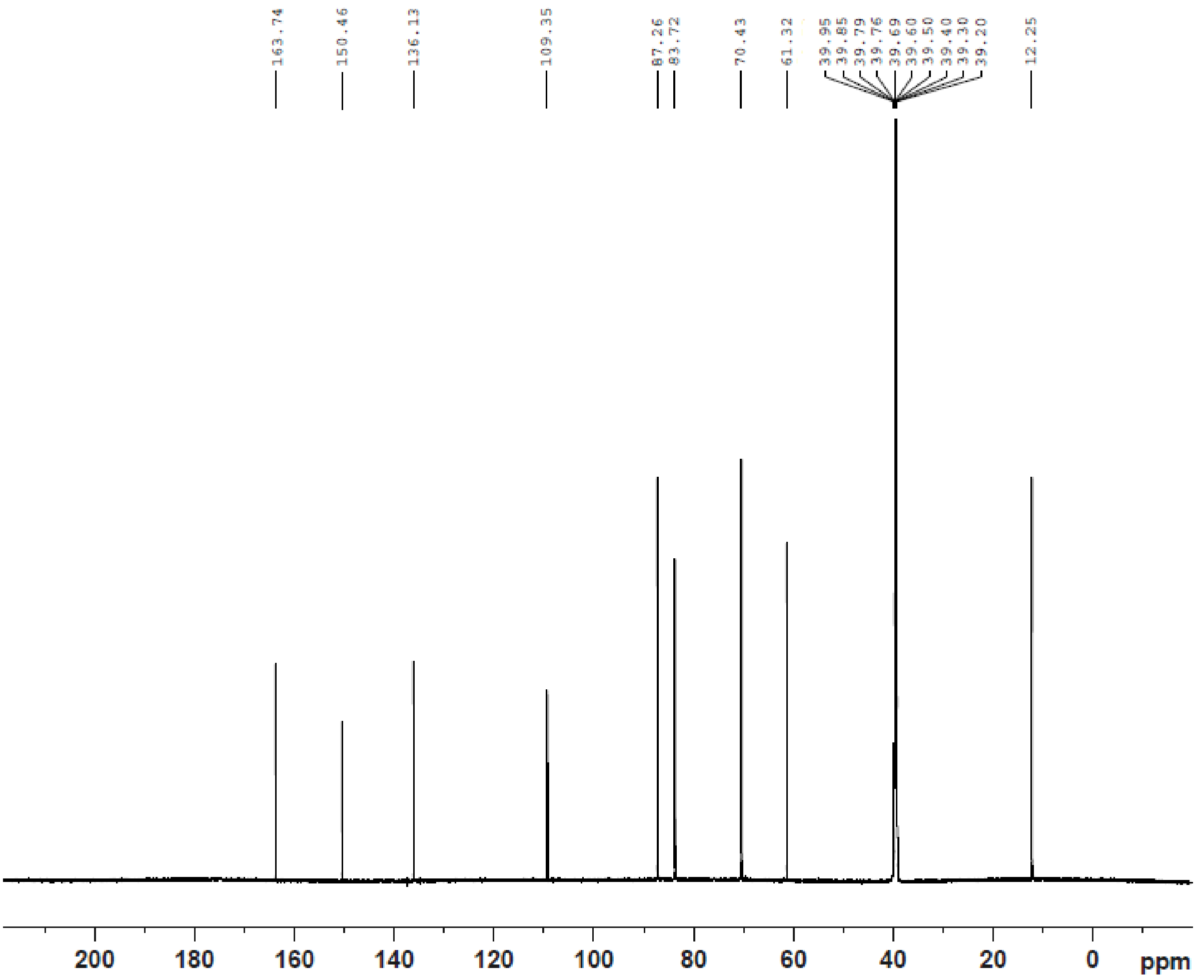

^{13}C NMR Spectrum of Compound **4** (DMSO-d_6)

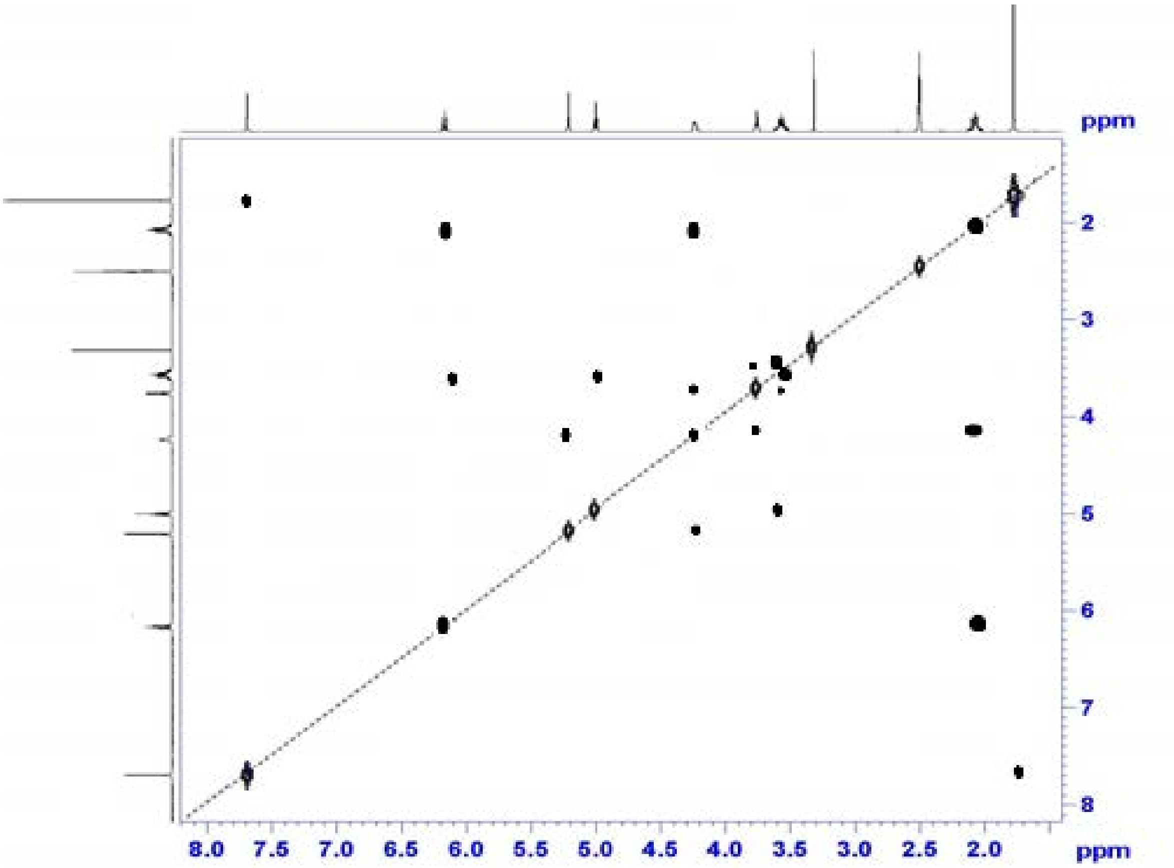

COSY Spectrum of Compound **4** (DMSO-d_6)

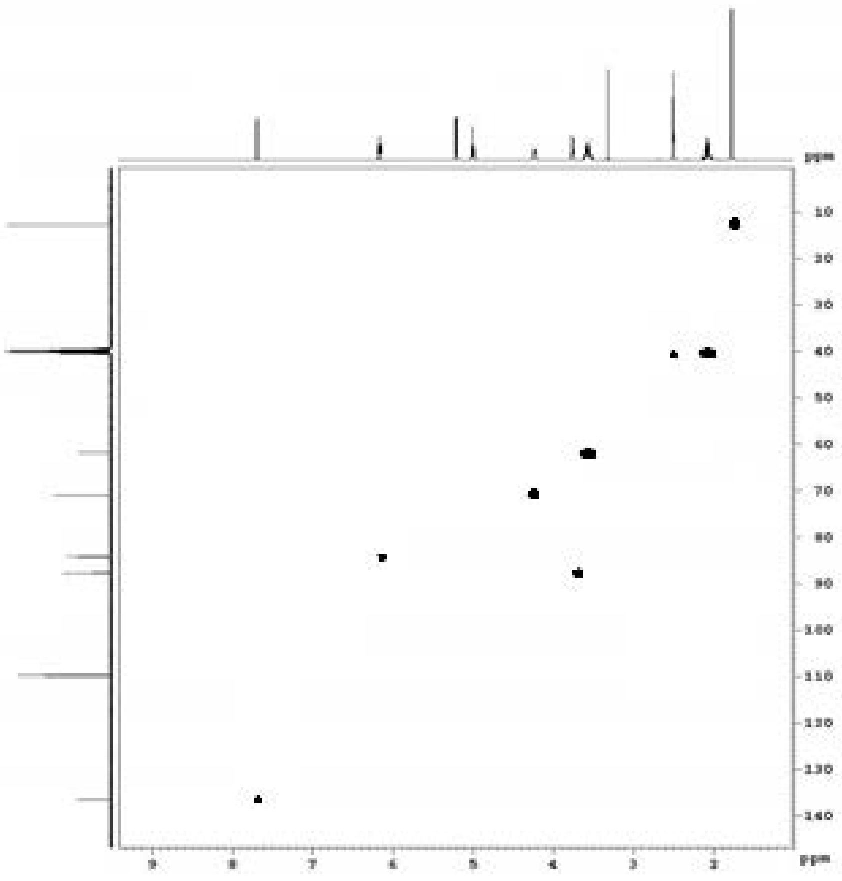

HSQC Spectrum of Compound **4** (DMSO-d_6)

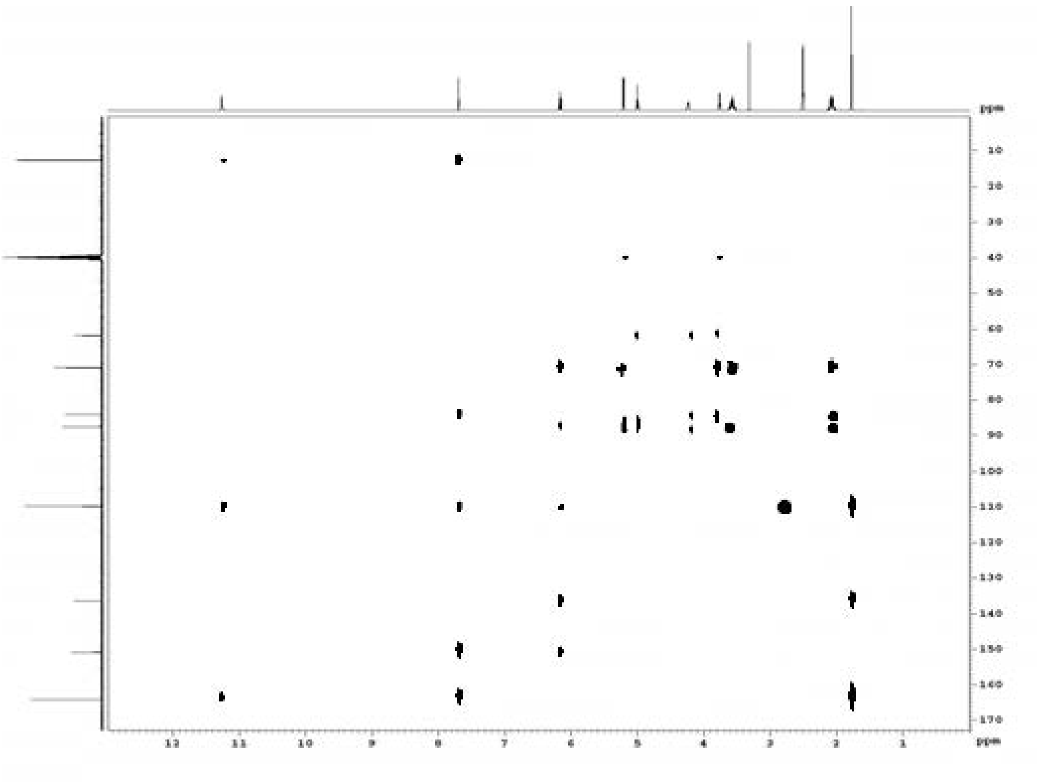

HMBC Spectrum of Compound **4** (DMSO-d_6)

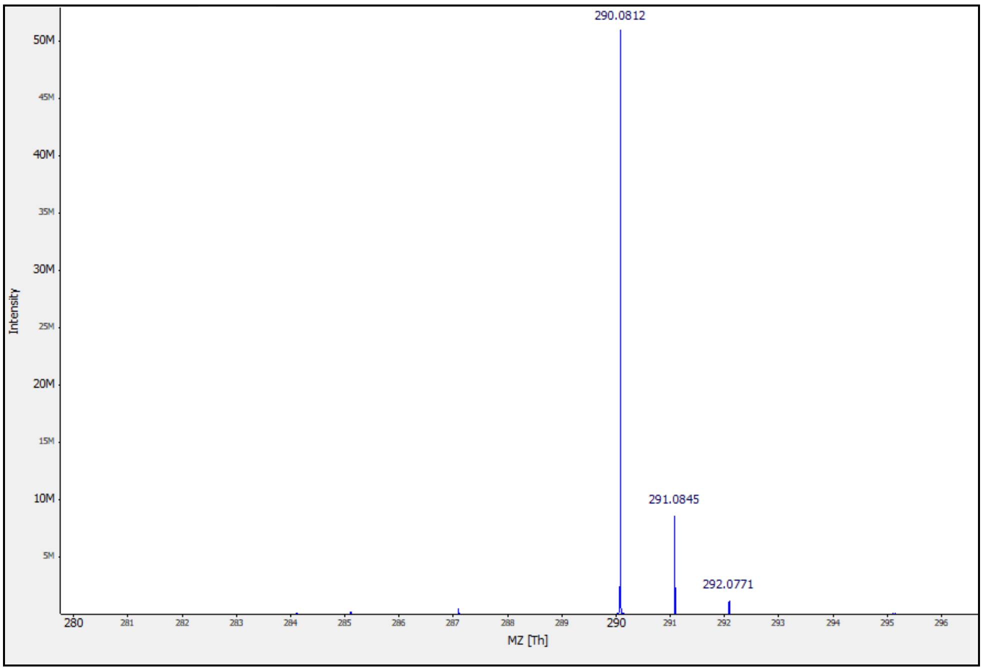

HRESIMS of Compound **4**.

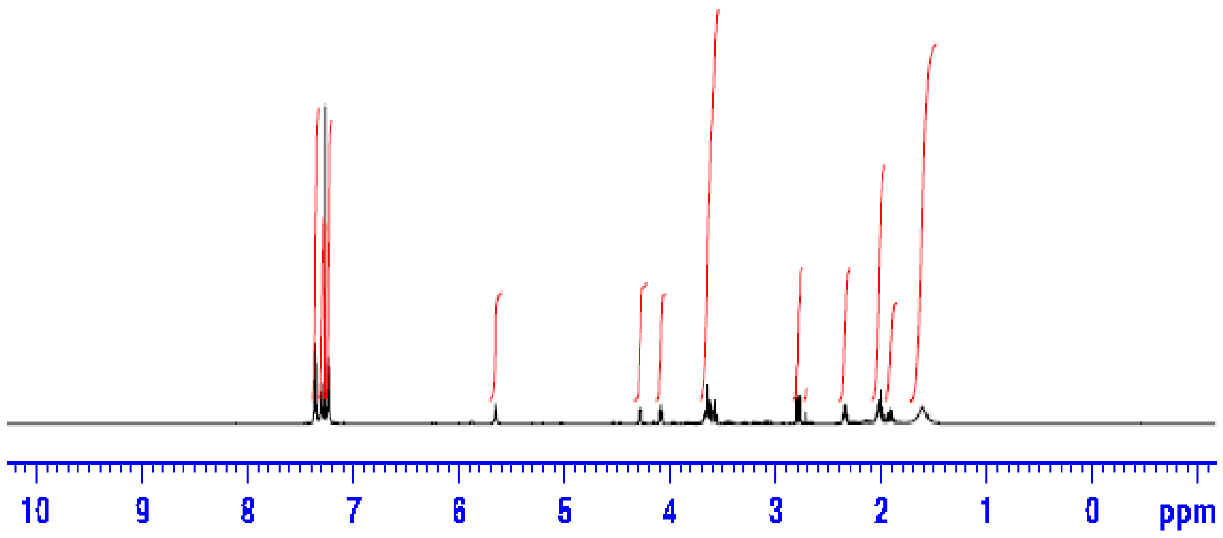

^{1}H NMR Spectrum of Compound **5** ($CDCl_3$).

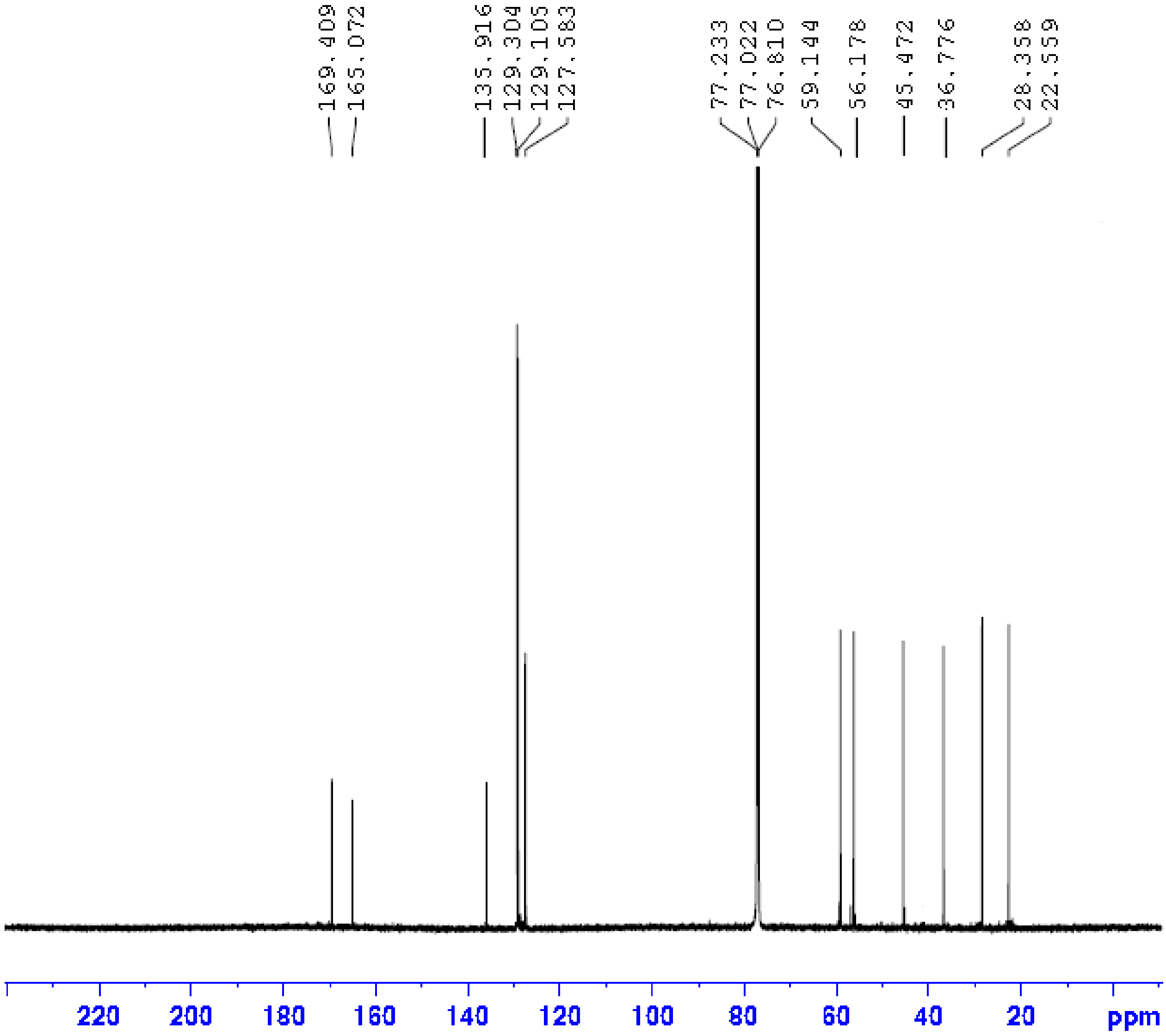

^{13}C NMR Spectrum of Compound **5** ($CDCl_3$).

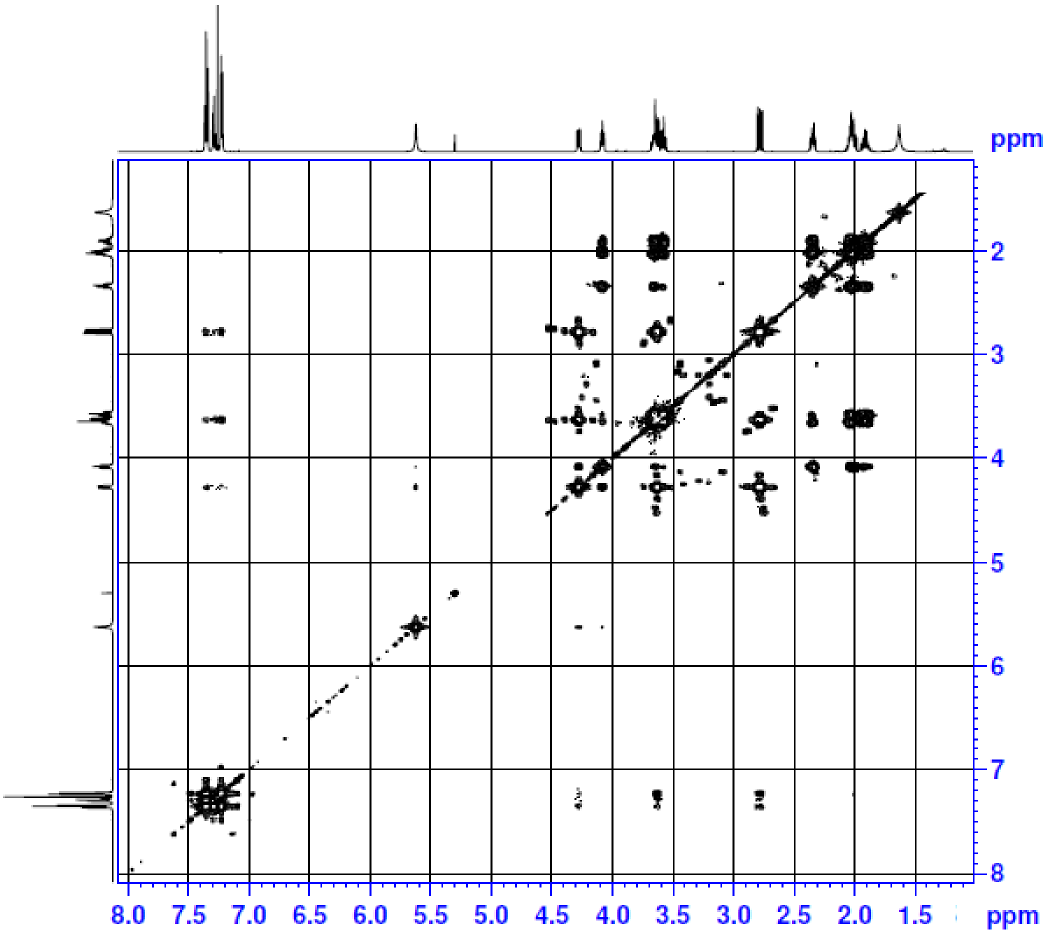

^{1}H-^{1}H COSY Spectrum of Compound **5** ($CDCl_3$).

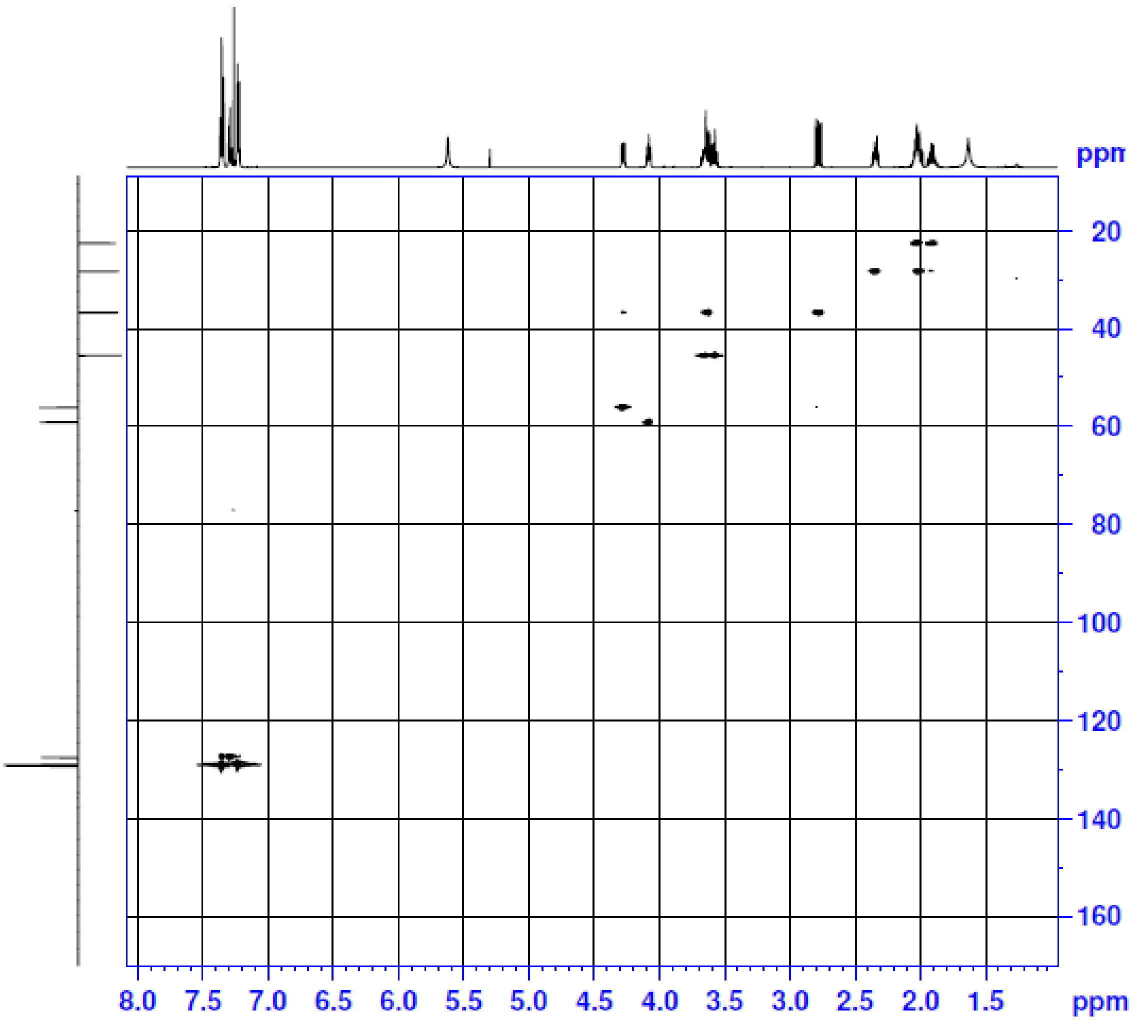

HSQC Spectrum of Compound **5** ($CDCl_3$).

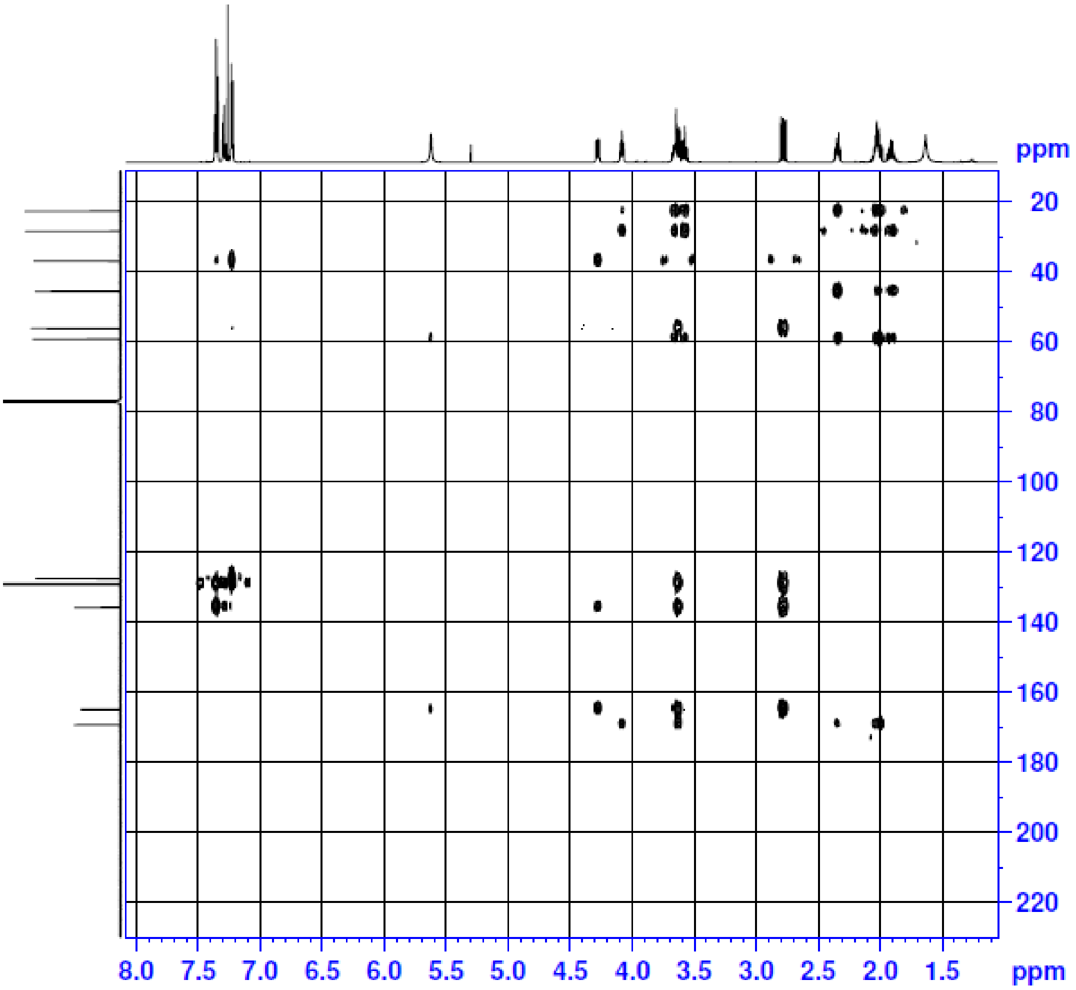

HMBC Spectrum of Compound **5** ($CDCl_3$).

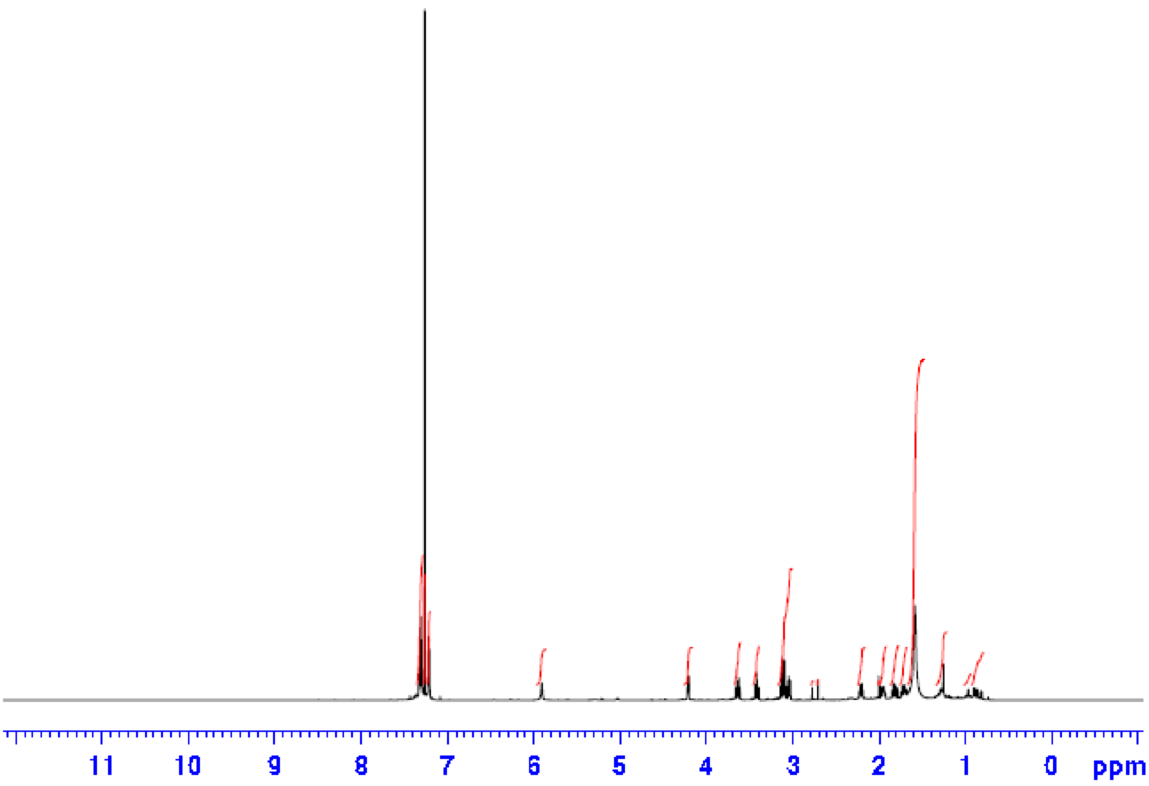

^{1}H NMR Spectrum of Compound **6** ($CDCl_3$).

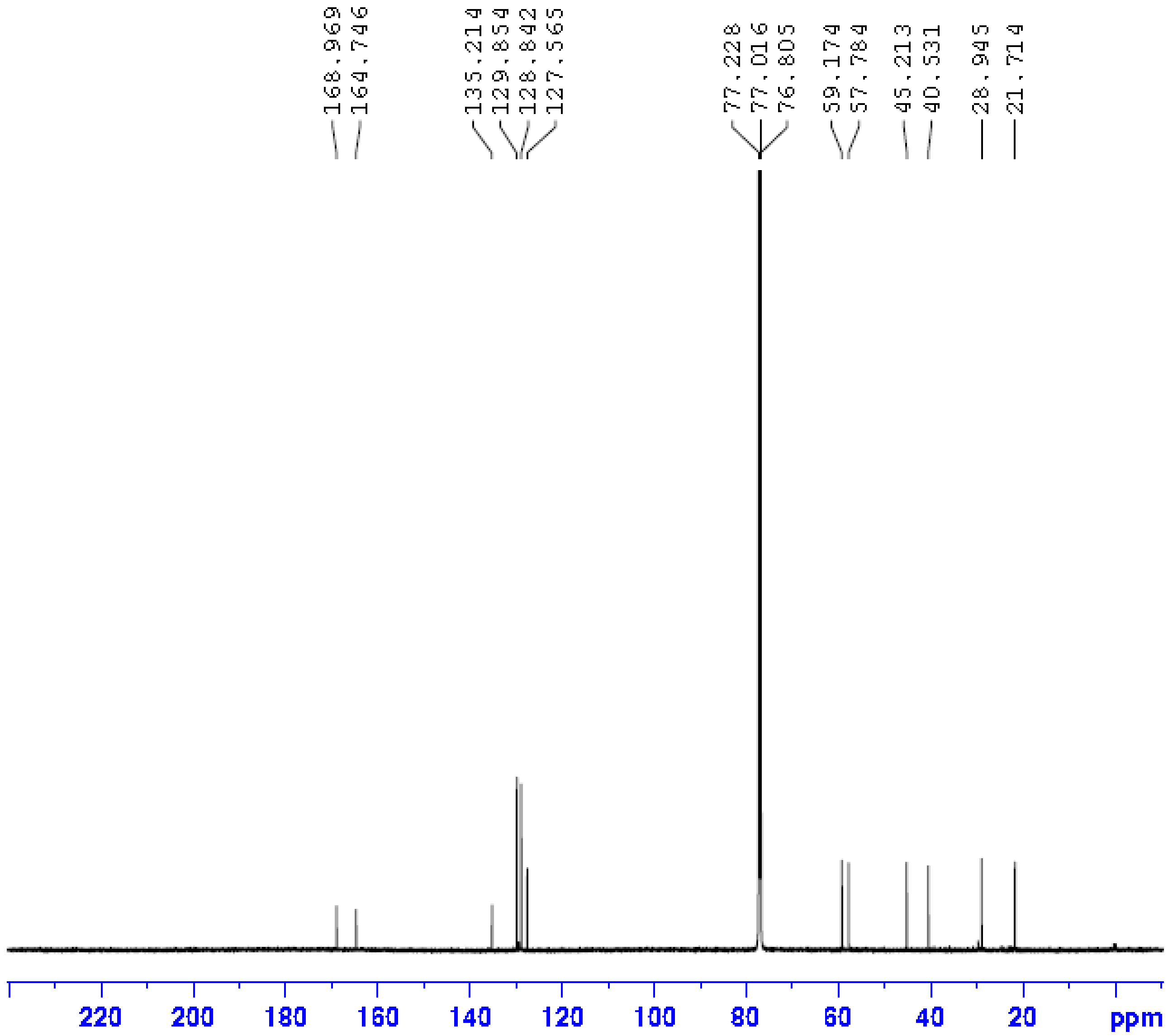

^{13}C NMR Spectrum of Compound **6** ($CDCl_3$)

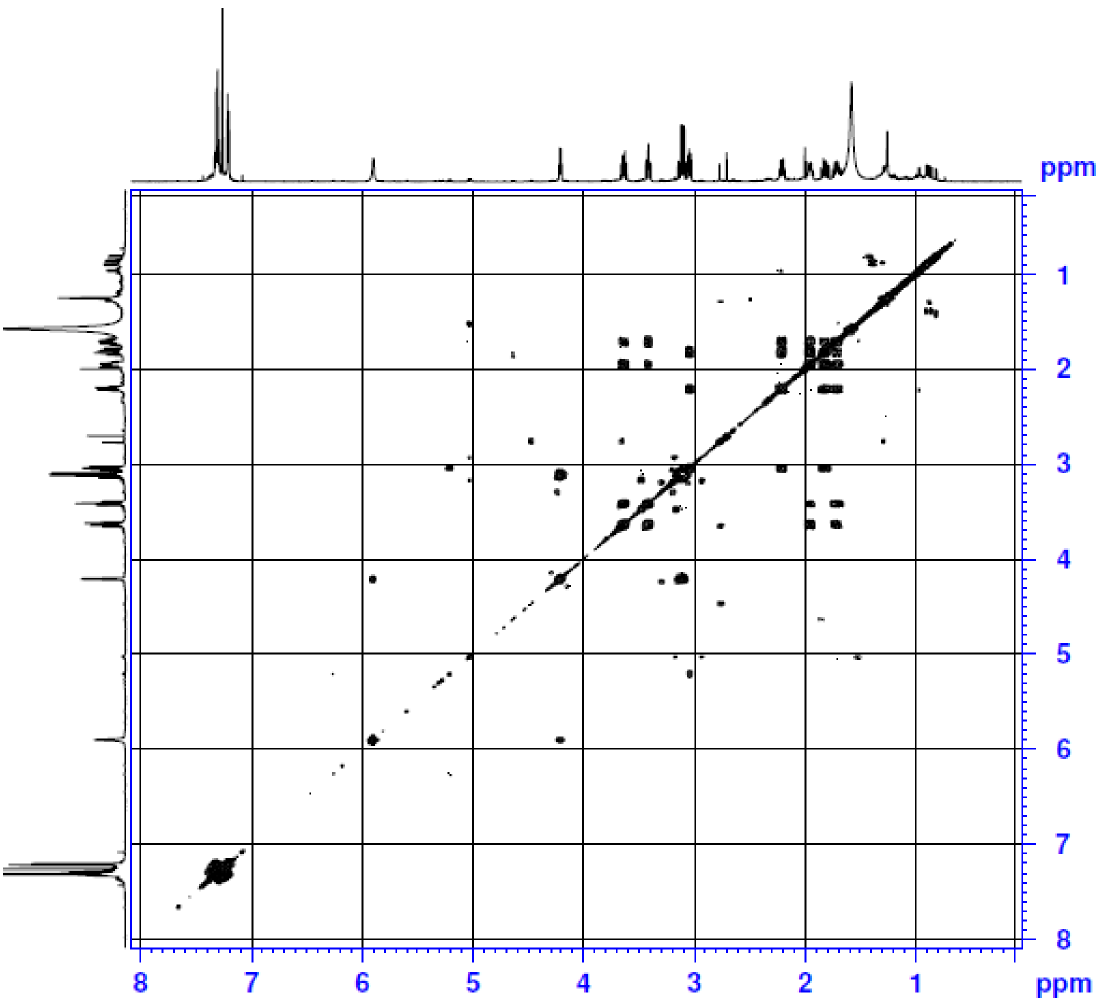

^{1}H-^{1}H COSY Spectrum of Compound **6** ($CDCl_3$).

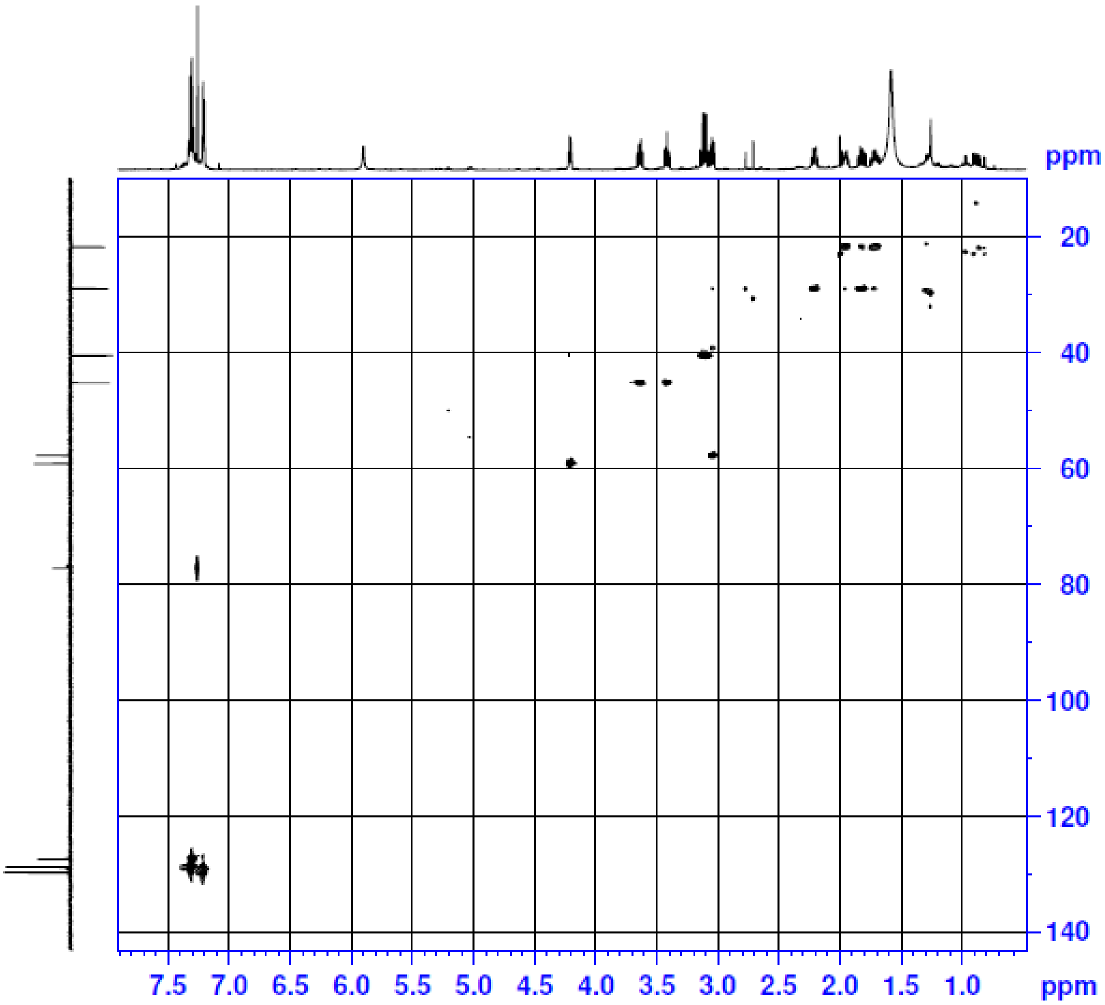

HSQC Spectrum of Compound **6** ($CDCl_3$).

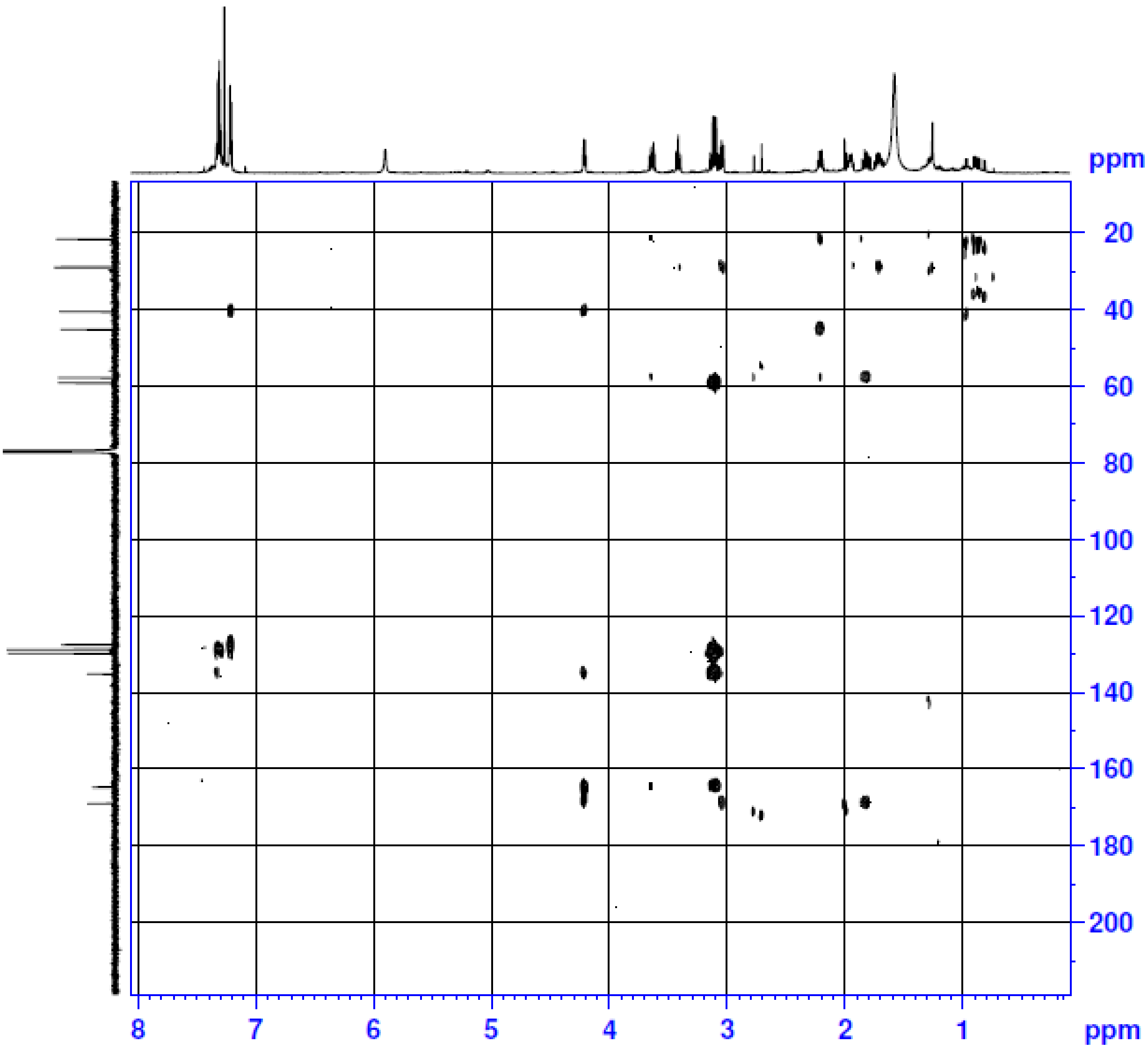

HMBC Spectrum of Compound **6** ($CDCl_3$).

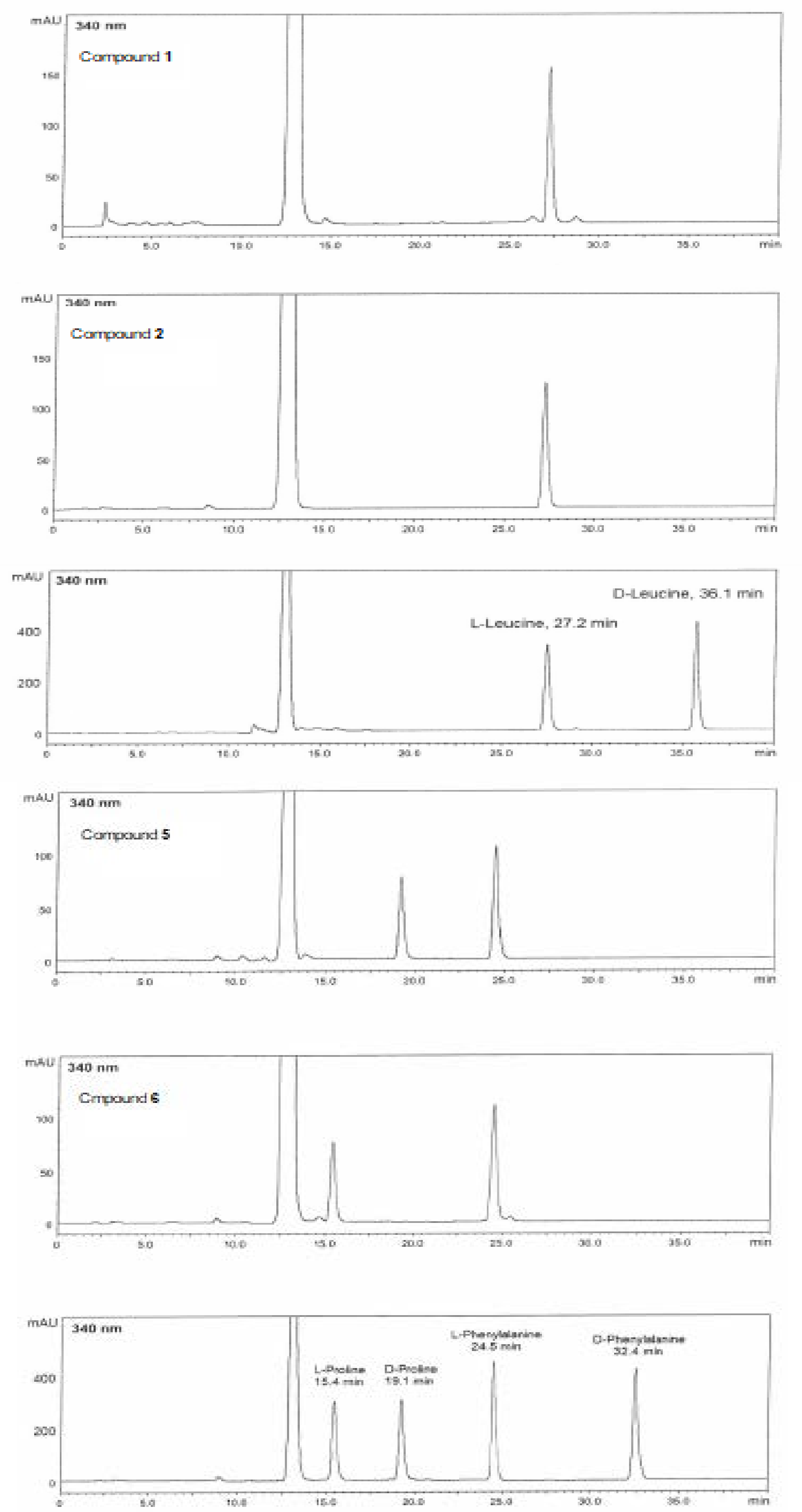

HPLC trace for Marfey's-derivatized hydrolysates of compounds **1**, **2**, **5, 6** and derivatized standard amino acids

Compound 1

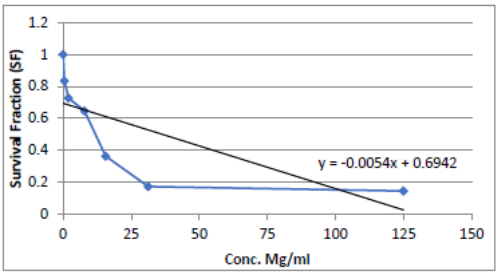

HCT116

Compound 1

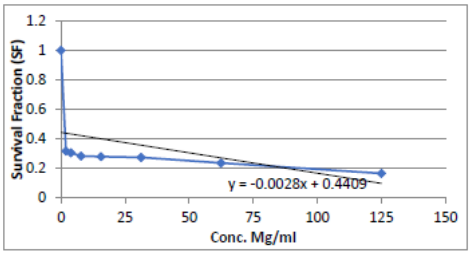

MCF7

Compound 2

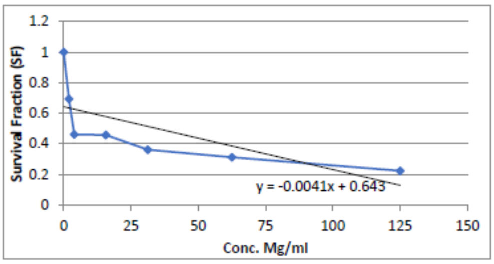

HCT116

Compound 2

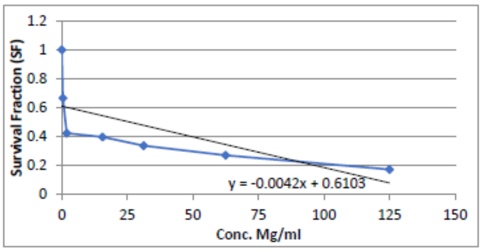

MCF7

Compound 3

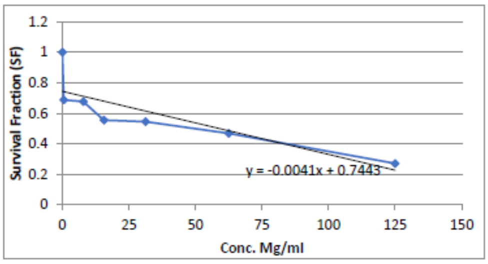

HCT116

Compound 3

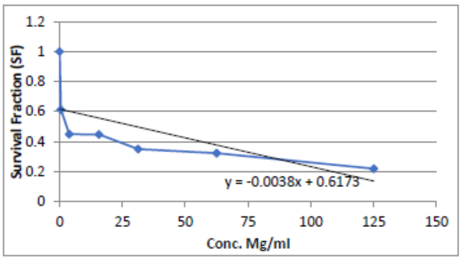

MCF7

Compound 4

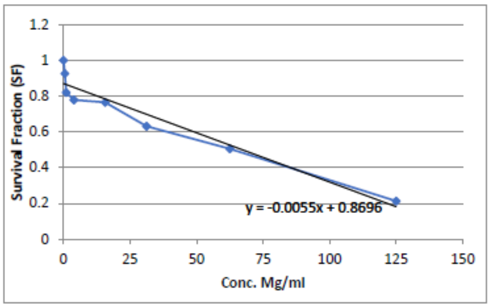

HCT116

Compound 4

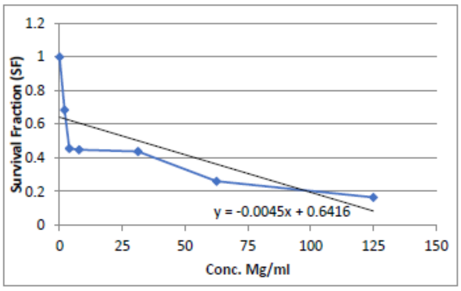

MCF7

Compound 5

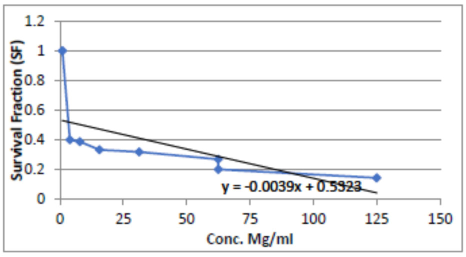

HCT116

Compound 5

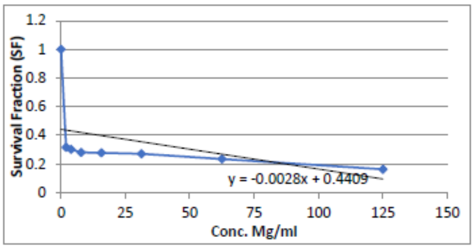

MCF7

Compound 6

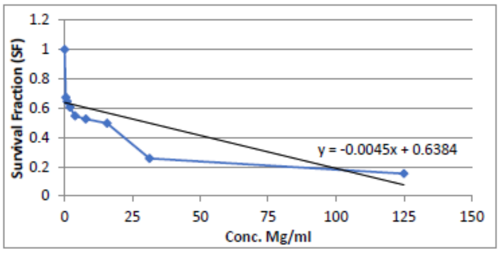

HCT116

Compound 6

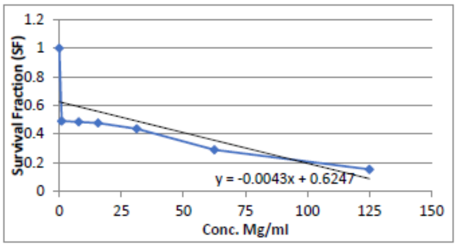

MCF7

Concentration-response profiles for compounds **1-6**.
